# Direct evidence for conformational dynamics in major histocompatibility complex class I molecules

**DOI:** 10.1074/jbc.M117.809624

**Published:** 2017-10-11

**Authors:** Andy van Hateren, Malcolm Anderson, Alistair Bailey, Jörn M. Werner, Paul Skipp, Tim Elliott

**Affiliations:** From the ‡ Institute for Life Sciences and Centre for Cancer Immunology, Faculty of Medicine,; the ¶ Centre for Proteomic Research, Biological Sciences, and Institute for Life Sciences, and; the ‖ Institute for Life Sciences, Centre for Biological Sciences, and Faculty of Natural and Environmental Sciences, University of Southampton, Building 85, Southampton SO17 1BJ and; the § Waters Corporation, Stamford Avenue, Altrincham Road, Wilmslow SK9 4AX, United Kingdom

**Keywords:** antigen presentation, hydrogen–deuterium exchange, major histocompatibility complex (MHC), protein conformation, protein dynamic, protein folding, protein structure, structure-function, allotype, hydrogen–deuterium exchange mass spectrometry

## Abstract

Major histocompatibility complex class I molecules (MHC I) help protect jawed vertebrates by binding and presenting immunogenic peptides to cytotoxic T lymphocytes. Peptides are selected from a large diversity present in the endoplasmic reticulum. However, only a limited number of peptides complement the polymorphic MHC specificity determining pockets in a way that leads to high-affinity peptide binding and efficient antigen presentation. MHC I molecules possess an intrinsic ability to discriminate between peptides, which varies in efficiency between allotypes, but the mechanism of selection is unknown. Elucidation of the selection mechanism is likely to benefit future immune-modulatory therapies. Evidence suggests peptide selection involves transient adoption of alternative, presumably higher energy conformations than native peptide–MHC complexes. However, the instability of peptide-receptive MHC molecules has hindered characterization of such conformational plasticity. To investigate the dynamic nature of MHC, we refolded MHC proteins with peptides that can be hydrolyzed by UV light and thus released. We compared the resultant peptide-receptive MHC molecules with non-hydrolyzed peptide-loaded MHC complexes by monitoring the exchange of hydrogen for deuterium in solution. We found differences in hydrogen–deuterium exchange between peptide-loaded and peptide-receptive molecules that were negated by the addition of peptide to peptide-receptive MHC molecules. Peptide hydrolysis caused significant increases in hydrogen–deuterium exchange in sub-regions of the peptide-binding domain and smaller increases elsewhere, including in the α3 domain and the non-covalently associated β_2_-microglobulin molecule, demonstrating long-range dynamic communication. Comparing two MHC allotypes revealed allotype-specific differences in hydrogen–deuterium exchange, consistent with the notion that MHC I plasticity underpins peptide selection.

## Introduction

Major histocompatibility complex class I (MHC I)^2^ molecules help protect jawed vertebrates from pathogens and cancer by selecting peptides, derived from the proteins present within virus-infected or malignant cells of the host, and presenting them at the cell surface, where they can be recognized by cytotoxic T lymphocytes.

MHC I molecules, which consist of a polymorphic heavy chain (HC) non-covalently associated with a monomorphic β_2_-microglobulin (β_2_m) molecule, become loaded with peptides in the endoplasmic reticulum (ER), following recruitment to the peptide-loading complex (PLC) by calreticulin (1). The PLC assembles around the peptide transporter associated with antigen presentation (2–4), to which MHC I molecules are tethered via the co-factor tapasin (5–7). The oxidoreductase ERp57 associates with both calreticulin and tapasin to complete a synergistic network of interactions (8–11). For efficient antigen presentation to ensue, each MHC I HC-β_2_m heterodimer must assemble with a peptide that complements the unique chemistry of the peptide-binding groove.

The intracellular peptide-selecting function of MHC I can be disrupted by introducing point mutations into the MHC I HC that prevent binding to tapasin (12) or by genetic ablation of tapasin (5, 6, 13–15). These studies showed that the ability to discriminate between peptides is an intrinsic property of MHC I molecules, which differs in efficiency between MHC I allotypes (16, 17), and that tapasin quantitatively and qualitatively enhances this peptide-selection function (10, 16, 18). Thus, some MHC I allotypes like the human leukocyte antigen (HLA) B*44:05 (16) and the chicken BF2*15:01 molecules (19, 20) are relatively efficient at selecting high-affinity peptide cargo in the absence of tapasin. This can be advantageous because the expression of tapasin can be down-regulated, as has been documented with a variety of tumors and which is generally associated with worse prognosis (21–24) or targeted by pathogen-encoded immune evasion molecules (25, 26). Conversely, other allotypes, like HLA-B*44:02 and BF2*19:01, are inefficient at loading peptide cargo in the absence of tapasin. Tapasin therefore normalizes this variation in intrinsic “peptide selector” function across different allotypes (17, 27). Consequently, MHC I allotypes with low intrinsic selector function are often described as being tapasin-dependent, whereas those allotypes with high intrinsic selector function are considered as being tapasin-independent, despite the fact that all MHC I allotypes benefit from tapasin to some degree (16).

The mechanism underpinning the intrinsic peptide-selecting function is not known. Molecular dynamic simulations suggest that in the absence of the stabilizing interactions provided by a bound peptide, the MHC I peptide-binding domain becomes disordered and that the adoption of such a non-native state (or states) differs between MHC I allotypes (28–30). By combining molecular dynamics simulations with computational models describing the *in vivo* peptide-selecting function of different MHC I allotypes, we have identified that the adoption of such an alternative peptide-receptive conformation (or conformations) is important for MHC I peptide selection (27). Such a possibility is consistent with earlier findings that observed peptide-binding rates are faster than are predicted from measured dissociation rates and equilibrium constants, suggesting that MHC I molecules may change conformation during peptide binding (31). Furthermore we have suggested that the “allotype-specific” parameter that determines the intrinsic peptide-selecting function of MHC I molecules is the rate at which they change from a peptide-receptive to a peptide-bound state (27, 32). Thus, allotypes like HLA-B*44:05 and BF2*15:01, which efficiently assemble with high-affinity peptides in the absence of tapasin, may fluctuate rapidly between conformations, thereby allowing more frequent sampling of potential peptide ligands.

Tapasin interacts with MHC I molecules via two sites, one in the MHC I α3 domain and a second in the peptide-binding domain (33–38), and in so doing tapasin accelerates the rates that peptides bind to and dissociate from MHC I molecules (10, 19, 39). We have suggested that tapasin uses both these sites of interaction to modulate the plasticity of MHC I molecules (19, 20, 27, 28, 40), such that the rate at which MHC I molecules transit between conformations, or states, is catalyzed, facilitating rapid peptide sampling. To date, most of the data supporting this model for peptide selection by MHC I and the role of tapasin are indirect (19, 39) or are derived from *in silico* experiments (20, 27, 35, 38), although direct observation by nuclear magnetic resonance spectroscopy of an empty and unstructured peptide-binding domain has been reported (41). More direct studies have been hindered by the instability of MHC I molecules in the absence of peptides (42). Although this is true for most MHC I molecules, we have observed that two closely related chicken MHC I allotypes, BF2*15:01 and BF2*19:01, are able to adopt and retain a peptide-receptive state, generated via UV-induced hydrolysis of the bound peptide ligand, to allow comparison with their non-hydrolyzed peptide-loaded counterparts.

In this report we refolded these two chicken MHC I allotypes with UV labile conditional ligands and compared them in their conditional ligand-loaded (non-hydrolyzed) and peptide-receptive (hydrolyzed) states by hydrogen–deuterium exchange mass-spectrometry (HDX-MS). With the exception of proline, all the protein backbone amide groups can exchange their hydrogen atoms with hydrogen atoms in the solvent or with deuterium atoms if a protein is transferred into a deuterated solution. The propensity of backbone amide hydrogens to exchange depends on the rate of proton exchange with the solvent and, under suitable conditions, may be limited by the opening of hydrogen bonds the nitrogen amide proton may be involved in. The opening rates of hydrogen bonds are measures of local backbone dynamics and may be modulated by a number of factors. Hydrogen–deuterium (H-D) exchange can be defined by mass spectrometry in a stop-flow-type experiment, where a rapid change of solvent, usually achieved by dilution, initiates a period of exchange, which can then be quenched by a change of pH. Digestion of the protein with a protease, such as pepsin, produces polypeptide fragments that can be separated by liquid chromatography and analyzed for proton and deuterium content by mass spectrometry.

Our novel combination of HDX-MS with conditional ligand-loaded MHC I molecules enabled us to make direct observations of the differences in conformational plasticity, as measured by HDX-MS, that have until now been predicted, or indirectly inferred, to occur in MHC I molecules in the peptide-loaded and peptide-receptive states. Specifically, we sought experimental evidence for the following: (*a*) an increase in conformational plasticity and its localization in MHC I molecules following conditional ligand hydrolysis; (*b*) differences in the dynamics of MHC I allotypes that vary in their intrinsic peptide-selecting functional abilities; and; (*c*) the range of communication of dynamic events between the peptide-binding domain and the membrane-proximal MHC I α3 domain and the non-covalently associated β_2_m molecule following conditional ligand hydrolysis.

## Results

### BF2 molecules remain receptive to binding peptides after conditional ligand hydrolysis for approximately 2 days

We have previously shown that two closely related chicken MHC I allotypes, BF2*15:01 and BF2*19:01, which differ by eight amino acids (Table 1) but share similar peptide binding specificities (43, 44), can be refolded with the same UV labile peptide ligand (conditional ligand hereafter) (19). UV-induced hydrolysis of the conditional ligand synchronously generates peptide-receptive MHC I molecules that bind the fluorescent peptide KRLIGK*RY (where K* denotes TAMRA-labeled lysine) with near identical affinity. However, these allotypes differ in their abilities to load peptide cargo in the absence of tapasin, in their affinities and on-rates with tapasin, and as substrates for tapasin-mediated peptide dissociation (19). These properties correlate with differences in their protein dynamic signatures evaluated *in silico* (20, 40), where we have noted similarities between the two chicken allotypes and the HLA B*44:02 and B*44:05 pair of allotypes, which may determine the extremes of tapasin dependence in humans (16, 17, 27). Specifically, we noted that the BF2*15:01 and HLA B*44:05 allotypes efficiently self-assemble with high-affinity peptides, and in molecular dynamic simulations they show transient minima of alternative conformations (*i.e.* they possess greater plasticity) in the absence of peptide. In comparison, the BF2*19:01 and HLA B*44:02 allotypes predominantly occupy a single energy minimum in molecular dynamic simulations (20, 27, 40) and require assistance from tapasin to select high-affinity peptides. We therefore reasoned that comparison of these two chicken allotypes in the peptide-loaded and peptide-receptive states might enable us to directly observe differences in conformational plasticity following conditional ligand hydrolysis and to compare the dynamic profiles of the two MHC I allotypes that may help to explain why they differ in their assembly properties.

**Table 1:** **BF2 amino acid polymorphisms** Amino acid polymorphisms between the BF2*15:01 and BF2*19:01 allotypes are tabulated. Residues are numbered from the first residue of the mature protein and indicate the domain, and the element of secondary structure, in which the polymorphism is located. The equivalent residue of the HLA B*44:02 and B*44:05 allotypes is tabulated, with the equivalent residue number of the mature protein indicated.

Residue	HC domain, secondary structure	BF2*15:01	BF2*19:01	HLA B*44:02/B*44:05
22	α1, β strand 2	Tyr	Phe	Phe-22
69	α1, α1-helix	Thr	Ser	Asn-70
79	α1, α1-helix	Thr	Ile	Thr-80
95	α2, β-strand 5	Leu	Trp	Arg-97
111	α2, β-strand 6	Ser	Arg	Asp-114
113	α2, β-strand 6	Asp	Tyr	B*44:02: D116
				B*44:05: Y116
126	α2, turn between β-strands 7–8	Asp	Gly	Asp-129
220	α3, β-strand 4	Gln	Arg	Gln-224

**Figure 1.**
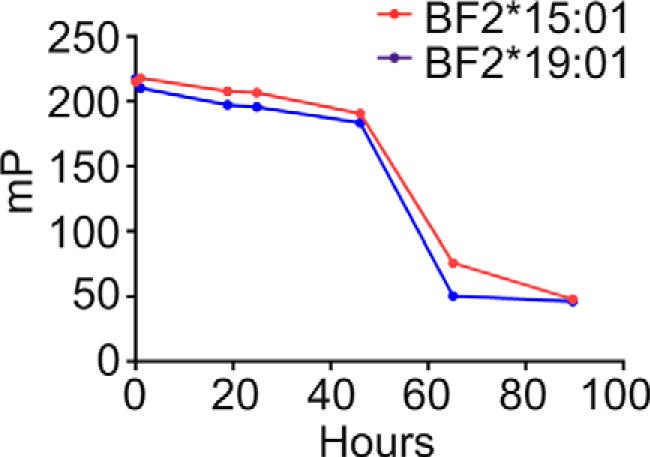
BF2*15:01 and BF2*19:01 remain receptive to binding KRLIGK*RY after conditional ligand hydrolysis for approximately 2 days. BF2*15:01 or BF2*19:01 conditional ligand-loaded complexes were UV-exposed on ice for 20 min before being incubated at 25 ºC. Aliquots were taken after various lengths of time and incubated with KRLIGK*RY for 1 h before peptide binding was measured by fluorescence polarization. Binding of fluorescent peptide is reported in millipolarization units. Unbound fluorescent peptide is assumed to have an mP level of 50. The data shown is one representative experiment from three experiments.

Characterization of the dynamic or biophysical properties of peptide-receptive MHC I molecules has been hindered by the propensity of MHC I molecules to aggregate and denature without the stabilizing influence of a high-affinity peptide. We found, however, that the BF2*15:01 and BF2*19:01 allotypes were receptive to peptide binding for up to 40 h following the synchronized hydrolysis of the conditional ligand (Fig. 1). In these experiments, we UV-exposed conditional ligand-loaded BF2*15:01 and BF2*19:01 complexes, and at various times, we sampled their ability to bind KRLIGK*RY. Fig. 1 shows that, as demonstrated previously (19), both BF2 allotypes bound KRLIGK*RY after UV-induced hydrolysis of the conditional ligand. The potentially heterogeneous occupancy of the peptide-binding grooves that might result from conditional ligand hydrolysis, where some or all of the molecules may be empty of peptide, or may retain one or both cleavage fragments of the conditional ligand, or may even contain the intact conditional ligand, is likely to reflect the mixed molecular nature of MHC I molecules assembling in the ER, where an initial low-affinity peptide cargo is thought to be iteratively replaced until a higher-affinity cargo is loaded (45, 46). Importantly, however, the UV-exposed samples retained the ability to bind KRLIGK*RY for approximately 2 days at 25 °C after UV exposure, with only 4% loss of binding sites for BF2*15:01 and 10% for BF2*19:01 after 24 h, rising to 12 and 16%, respectively, after 46 h. Both allotypes underwent a rapid loss of peptide-receptivity thereafter, with the polarization level measured after 65 h of incubation being 34% of the initial level for BF2*15:01 and 22% of the initial level for BF2*19:01. This decline in available binding sites presumably reflects unfolding or aggregation of the BF2 proteins. We have previously found KRLIGK*RY dissociates from these allotypes with a half-time of 0.6 h (BF2*15:01) or 1.7 h (BF2*19:01) (19), suggesting that the high polarization levels observed during the first 46 h represents an equilibrium in which KRLIGK*RY efficiently binds to and dissociates from the BF2 molecules. Thus, the ability of these MHC I allotypes to remain receptive to binding peptide for approximately 2 days after UV exposure offered the opportunity to characterize these two allotypes in the peptide-bound and peptide-receptive states.

### Conditional ligand hydrolysis leads to changes in hydrogen–deuterium exchange, which can be reversed by peptide binding

Having generated peptide-loaded and peptide-receptive MHC I proteins, we first asked whether conditional ligand hydrolysis might change the dynamics of MHC I molecules, as measured by HDX-MS, and whether any changes can be reversed by the binding of peptide. We have previously found that BF2*19:01 binds KRLIGK*RY peptide and undergoes dissociation more slowly than BF2*15:01 (19); therefore, we reasoned that this allotype would be the most stringent choice for these experiments. Conditional ligand-loaded BF2*19:01 complexes were either 1) UV-exposed, 2) not UV-exposed, or 3) UV-exposed and then supplemented with excess high-affinity peptide. H-D exchange was then allowed to proceed before the reaction was quenched, and the proteins were digested into polypeptides, separated by liquid chromatography, and analyzed by mass spectrometry. We achieved comprehensive coverage by obtaining 112 polypeptides derived from the HC (98.5% coverage, with an average redundancy of 5.95 polypeptides covering each residue that was included in the polypeptide map coverage, see supplemental Fig. S1*a*) and 40 polypeptides derived from β_2_m (100% coverage, average redundancy of 5.46, see supplemental Fig. S1*b*).

**Figure 2.**
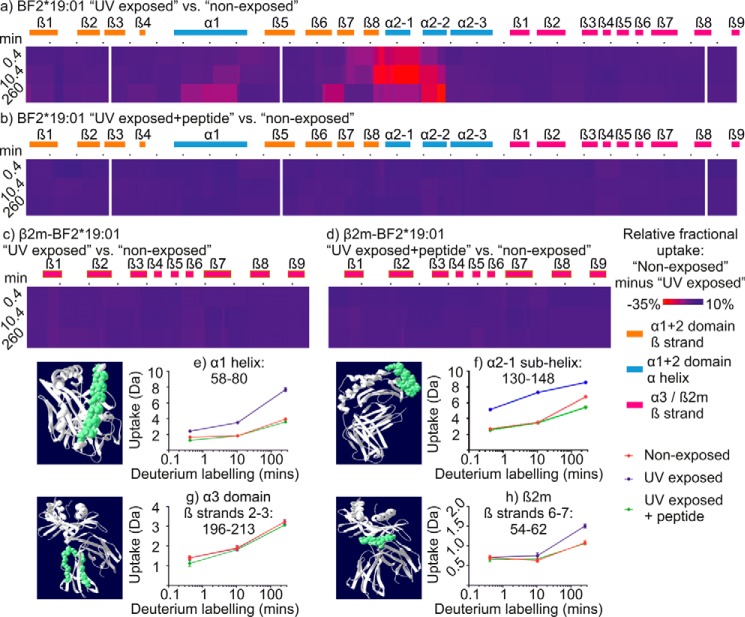
**Conditional ligand hydrolysis leads to changes in hydrogen–deuterium exchange, which can be reversed by peptide binding.** The hydrogen–deuterium exchange profiles of conditional ligand-loaded BF2*19:01 complexes that were not UV-exposed are compared with their UV-exposed counterparts (BF2*19:01 HC in *a* and β*_2_m* in *c*) or their UV-exposed and peptide-supplemented counterparts (BF2*19:01 HC in *b* and β*_2_m* in *d*). The relative fractional differences in deuterium uptake between UV-exposed samples and non-exposed samples (*a* and *c*) or between UV-exposed and peptide-supplemented samples and non-exposed samples (*b* and *d*) are shown as a heat map on a *red-blue* scale. *Gaps* in the heat maps indicate gaps in the polypeptide coverage. The *y* axis denotes the duration of the incubation in deuterated equilibrium buffer (in minutes). The *x* axis denotes the protein sequence, with every 10th residue indicated with a *period* and α-helices and β-strands indicated by *rectangles* (colored as in the key) and numbered sequentially according to their location in the protein sequence (see Fig. 3). Where overlapping polypeptides covered any given residue (average redundancy was ∼5–6, see supplemental Fig. 1 for polypeptide coverage maps), the deuterium uptake data were determined by the shortest polypeptide. Where multiple polypeptides are of the same shortest length, the polypeptide with the residue closest to the protein C terminus was used to determine the deuterium uptake data. The N terminus of each polypeptide was not used to render the heat map. *e–h* show deuterium uptake charts for the indicated polypeptides in which the mean average deuterium uptake is plotted for each exposure time and protein state, with the standard deviation that was observed between replicates represented using *vertical error bars*. Note that the scale of the *y* axes (deuterium uptake) differs between the polypeptides because of differences in the length of polypeptides and in the ability of backbone amide hydrogens to exchange with deuterium. For each polypeptide, the indicated sequence is shown in *green* based on a homology model of the BF2*15:01–KRLIGKRY complex.

**Figure 3.**
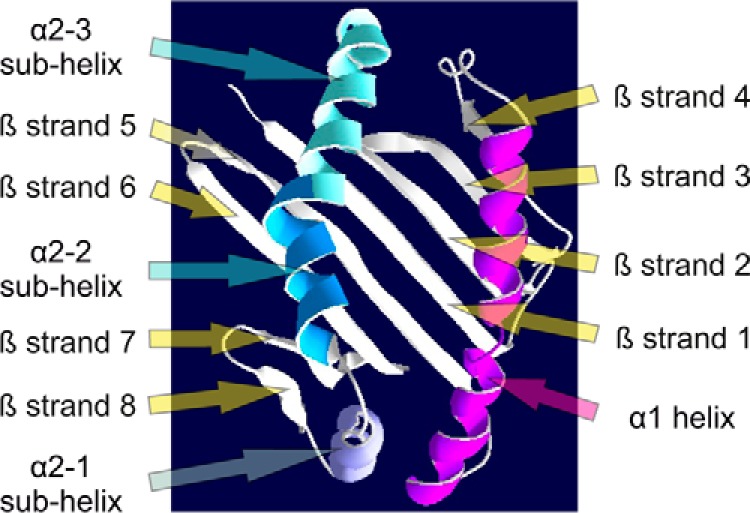
Model of the peptide-binding domain of BF2*15:01. The peptide-binding domain of BF2*15:01 (residues 1–176 of the mature protein) is shown modeled upon the BF2*21:01 structure (57) and viewed from above. The α-helices are labeled and colored in *red* (α1) or shades of *blue* (α2). The β-strands are numbered sequentially and labeled.

Fig. 2 shows the differences when the H-D exchange profile of UV-exposed BF2*19:01 molecules was compared with that of conditional ligand-loaded molecules that were not UV-exposed (Fig. 2, *HC* in *a* and β*_2_m* in *c*). In general terms, there was greater H-D exchange after UV exposure in the α1 and α2 domains than was observed in the α3 domain or the β_2_m molecule. The MHC I α1 and α2 domains combine to form the peptide-binding domain in which the peptide is partially buried, held by a network of interactions between two α-helices above eight antiparallel β-strands (Fig. 3). Therefore, the increased H-D exchange observed in the peptide-binding domain after UV exposure suggests that the rate of hydrogen bond opening was increased in the absence of an intact peptide. Crucially these increases in deuterium uptake were not observed when BF2*19:01 molecules were supplemented with high-affinity peptide immediately after UV exposure, which produced a similar exchange profile to their non-exposed conditional ligand-loaded counterpart (Fig. 2, *HC* in *b*, β*_2_m* in *d*, and see *e–h*). This confirms that the increased H-D exchange that is observed after UV exposure occurs in peptide-receptive, and not inactive, or aggregated MHC I molecules.

**Figure 4.**
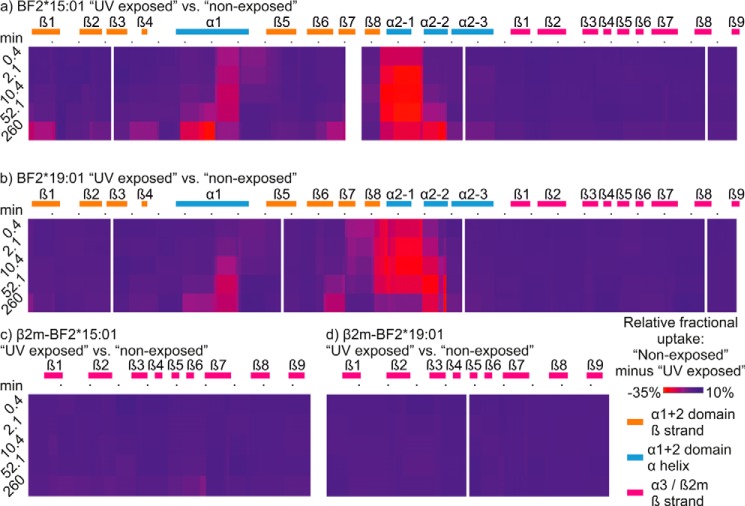
Conditional ligand hydrolysis changes the hydrogen–deuterium exchange profiles of BF2*15:01 or BF2*19:01 complexes. The hydrogen–deuterium exchange profiles of UV-exposed BF2*15:01 HC (*a*), BF2*19:01 HC (*b*), or the β_2_m molecules bound to each allotype (*c* and *d*) are compared with their non-exposed counterparts and presented as in Fig. 2, *a* and *c*.

Having ascertained that H-D exchange differs between UV-exposed peptide-receptive BF2*19:01 molecules and conditional ligand-loaded BF2*19:01 molecules that were not exposed to UV light, we next considered how the two MHC I allotypes might differ in their H-D exchange profiles. We therefore measured H-D exchange in the conditional ligand-loaded and peptide-receptive states for both allotypes (Fig. 4). The polypeptide coverage of both allotypes was comprehensive and we obtained the following: 44 polypeptides derived from β_2_m associated with the BF2*15:01 complex (100% coverage, average redundancy 5.81); 38 polypeptides derived from β_2_m associated with the BF2*19:01 complex (100% coverage, average redundancy 5.23); 119 polypeptides derived from the BF2*15:01 HC (96.7% coverage, average redundancy 6.41); and 105 polypeptides derived from the BF2*19:01 HC (98.5% coverage, average redundancy 5.55, supplemental Fig. S1). Of these polypeptides, 72 were common to both HC proteins (based upon their location within the protein sequence), and 30 polypeptides were derived from both β_2_m proteins. In the following analysis we consider these common polypeptides (deuterium uptake charts are available in supplemental Figs. S2–S5). Importantly, we found that UV exposure affected the exchange of backbone amide hydrogens of both allotypes in a similar overall fashion, with greater H-D exchange occurring in the α1 and α2 domains than in the α3 domains or the β_2_m molecules (Fig. 4). For both allotypes, we observed that for specific sub-regions, such as the α2–1 sub-helix, there was a greater difference in H-D exchange between the UV-exposed and non-exposed samples after incubation in deuterated buffer for 10.4–52.1 min than there was with the longest labeling period (shown as *deeper shades of red* in Fig. 4, *a* and *b*). The deuterium uptake charts of polypeptides derived from these sub-regions (for example, polypeptide 130–148, *panel AM* in supplemental Fig. S4) reveal that although UV-induced peptide hydrolysis resulted in a rapid and substantial increase in the rate of H-D exchange, with deuterium uptake continuing to increase at a slower rate thereafter, for the conditional ligand-loaded complexes that were not exposed to UV light the increase in H-D exchange occurred more slowly but continued throughout the labeling period. This resulted in the difference in uptake between UV-exposed and non-exposed states being the greatest after deuterium labeling for 10–52 min. Although there were many similarities between the two allotypes in their H-D exchange profiles, we also observed differences in the rates of H-D exchange in specific sub-regions of the two allotypes. We first considered the similarities and the differences in H-D exchange that were apparent between the two allotypes in their conditional ligand-loaded, non-exposed states.

### Localized allotype-specific differences in the uptake of deuterium in the conditional ligand-loaded state

**Figure 5.**
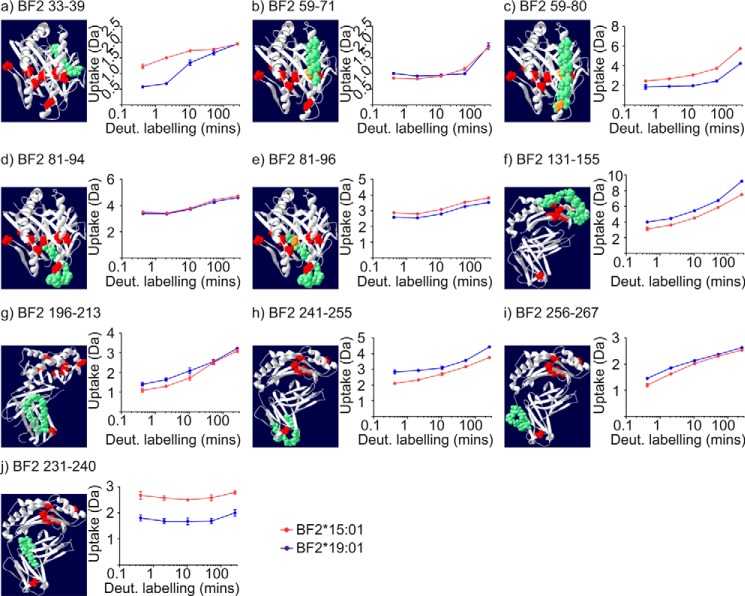
**Localized allotype-specific differences in the uptake of deuterium in the conditional ligand-loaded state.** *a–j*, comparison of the uptake of deuterium for selected polypeptides derived from both BF2 allotypes in the conditional ligand-loaded state (*i.e.* when hydrogen–deuterium exchange was initiated without prior UV exposure). The mean average deuterium uptake from the three replicates for each exposure time are shown, with the standard deviation between replicates represented using *vertical error bars*. For each polypeptide, the indicated sequence is shown in *green* based on a homology model of the BF2*15:01–KRLIGKRY complex. Polymorphic residues are shown in *red* or in *orange* when the polymorphism is located within the polypeptide of interest.

We found that for most polypeptides derived from the HC molecules of the conditional ligand-loaded complexes there was no significant difference in H-D exchange between the allotypes (supplemental Fig. S2). This was also the case for all of the polypeptides derived from the β_2_m molecules associated with the non-exposed MHC complexes (supplemental Fig. S3). However, Fig. 5 shows there were seven HC sub-regions where H-D exchange differed between the two allotypes.

Most of the sub-regions where H-D exchange differed between the allotypes were within the peptide-binding domain, where seven of the eight polymorphic residues are located (Table 1). In the α1 domain there were two sub-regions where faster uptake of deuterium was observed for polypeptides derived from the BF2*15:01 allotype (Fig. 5, *a–c*). The first were residues 33–39, which forms most of β-strand 3 (Fig. 5*a*). The differential uptake of deuterium observed for this polypeptide may be related to the nearby polymorphic residue at position 22, located in the neighboring β-strand (Table 1). This polymorphic residue may influence the ionic or hydrogen-bonding interactions of residues 33–39 or influence their solvent accessibility such that there is faster H-D exchange in the context of Tyr-22 (BF2*15:01) compared with Phe-22 (BF2*19:01). The second sub-region involved residues 71–80, which encode the second half of the α1-helix (compare Fig. 5, *b* and *c*). Here, differences in the rate at which deuterium was incorporated into residues 71–80 may reflect an influence on the dynamics of this portion of the α1-helix caused by either or both of the polymorphisms located within the α1-helix at positions 69 and 79.

In the α2 domain there was modestly greater uptake of deuterium into residues 95 and 96 of the BF2*15:01 allotype, which are situated in the center of β-strand 5 (compare Fig. 5, *d* and *e*). This differential uptake may reflect that residue 95 is polymorphic, with there being slightly greater exchange of backbone amide protons in the context of Leu-95 (BF2*15:01) as opposed to Trp-95 (BF2*19:01). In contrast, however, we observed faster H-D exchange for residues 130–155 derived from the conditional ligand-loaded BF2*19:01 allotype (polypeptide 131–155 is shown in Fig. 5*f*, which encodes the α2–1 sub-helix, parts of β-strand 8, and the α2–2 sub-helix, and their connecting residues, but see also supplemental Fig. S2 for alternative polypeptides). The difference between the allotypes in H-D exchange involving these regions may reflect variations in their local environment caused by the polymorphic residues at positions 111 and 113 situated beneath the α2–2 sub-helix in β-strand 6, which are located next to or within, respectively, the F pocket of the peptide-binding groove.

In the α3 domain there is a single polymorphic residue at position 220 in β-strand 4. This β-strand is of interest because mutation of residues 222 or 227 in mammalian MHC I molecules (equivalent to BF2 residues 218 and 223) have been shown to impair association with tapasin and the PLC and to impair peptide selection (37, 47, 48). We were unable to determine whether any of the polypeptides containing this polymorphic residue differed in their rates of H-D exchange, because all the deuterium that they incorporated occurred within the shortest labeling period (supplemental Fig. S2). However, we observed allotype-specific differences in H-D exchange in two nearby sub-regions. For residues 196–213 (Fig. 5*g*) and residues 241–267 (Fig. 5, *h* and *i*), there was faster uptake of deuterium into the polypeptides derived from the BF2*19:01 allotype, which may be indirectly caused by interactions with the polymorphic residue at position 220.

Interestingly, polypeptide 231–240, which encodes most of β-strand 6, half of β-strand 7, and their connecting β-hairpin turn and which underlies the floor of the peptide-binding groove (Fig. 5*j*), produced a contrasting pattern of allotype-specific differences in H-D exchange to that of the contiguous polypeptide 241–255 (Fig. 5*h*). Specifically, we observed greater H-D exchange in polypeptide 231–240 derived from the BF2*15:01 allotype, whereas for polypeptide 241–255 H-D exchange occurred faster in the BF2*19:01 molecule. As there are no polymorphic residues in close proximity to residues 231–240, this difference between allotypes may reflect subtle differences in the secondary structures of the two allotypes. For example, there may be one fewer hydrogen bond involving residues 231–240 in BF2*15:01 compared with BF2*19:01, meaning there is a difference in the number of backbone amide protons that are amenable to exchange.

Therefore, our comparison of both allotypes in their conditional ligand-loaded states revealed that although the uptake of deuterium did not differ between allotypes for most sub-regions of the proteins, there were several localized differences in deuterium uptake between the two allotypes, which may reflect the direct or indirect influence of nearby polymorphic residues or indicate subtle differences in secondary structure.

### Sub-regions of the peptide-binding domain show the fastest increases in H-D exchange following conditional ligand hydrolysis and differ between allotypes

We next characterized how conditional ligand hydrolysis affected the dynamics of the two allotypes, as measured by HDX-MS (Fig. 4). We observed that whereas both allotypes share an overall pattern of increased H-D exchange in their peptide-receptive states, there were numerous examples of localized allotype-specific differences in the rates of H-D exchange.

### α1 domain-encoded β-strands

The α1 domain encodes one-half of the peptide-binding groove and consists of an α-helix, β-strands 1–4, and the residues that connect these elements of secondary structure. The α1-helix forms one wall of the peptide-binding groove and runs above the four β-strands that form part of the floor of the peptide-binding groove, which are situated toward the end of the groove that binds the peptide N terminus (Fig. 3).

**Figure 6.**
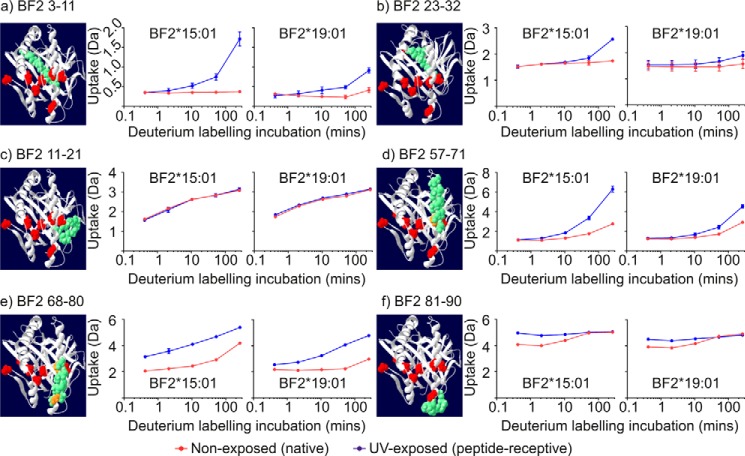
**Deuterium uptake charts for polypeptides derived from the α1 domains.** *a–f*, deuterium uptake charts for the indicated polypeptides derived from the α1 domains. The mean average deuterium uptake is plotted for each exposure time and protein state, with the standard deviation that was observed between replicates represented using *vertical error bars*. For each polypeptide, the indicated sequence is shown in *green* based on a homology model of BF2*15:01. Polymorphic residues are shown in *red* or in *orange* when the polymorphism is located within the polypeptide of interest.

Analysis of polypeptides that are derived entirely, or almost completely, from α1 domain-encoded β-strands reveals little incorporation of deuterium in the absence of UV exposure (*red lines,* Fig. 6*a*, polypeptide 3–11 covering most of the first β-strand, and Fig. 6*b*, polypeptide 23–32 constituting most of the second β-strand, a short intervening β-hairpin turn, and part of the third β-strand). Following conditional ligand hydrolysis there was increased H-D exchange for both allotypes, which first became apparent after incubation in deuterium for 10 min and became more prominent with longer deuterium labeling incubations (*blue lines,* Fig. 6, *a* and *b*). The greater uptake of deuterium that was observed following conditional ligand hydrolysis indicates that the local environment of these β-strands was significantly changed and that in the peptide-receptive state there was faster opening of hydrogen bonds and consequently faster H-D exchange than in the conditional ligand-loaded state. Notably, there was faster and, at least during these deuterium labeling periods, greater uptake of deuterium in polypeptides derived from β-strands of peptide-receptive BF2*15:01 molecules.

Other polypeptides that were also derived from α1 domain-encoded β-strands, but which included a larger proportion of the connecting β-turns, showed a different H-D exchange profile. As exemplified by polypeptide 11–21 (Fig. 6*c*), which constitutes the end of the first β-strand, a seven-residue β-hairpin turn, and the start of the second β-strand, there was increased uptake of deuterium throughout the labeling incubation, which occurred irrespective of UV exposure. This greater uptake of deuterium is consistent with the fact that in contrast to the underlying β-strands, this region is solvent-exposed and might be expected to display extensive and rapid H-D exchange.

### α1-helix

Analysis of the various polypeptides derived from the α1-helix revealed an exchange profile that combined some of the features of α1 domain-encoded β-strands with some of the properties of the connecting β-turns. As observed with the connecting β-turns, deuterium was incorporated into the α1-helix throughout the labeling period, even without prior UV exposure (Fig. 6*d*, polypeptide 57–71, which encode most of the first five turns of the helix, starting from the end of the peptide-binding groove that binds the peptide N terminus, and Fig. 6*e*, polypeptide 68–80, which constitute nearly four complete turns starting from the center and extending toward, but does not include, the last turn of the α1-helix, which is located toward the end of the peptide-binding groove that binds the peptide C terminus). This suggests that some of the hydrogen bonds of the α1-helix of conditional ligand-loaded molecules open faster than is apparent for the β-strands of the floor, and consequently there is a greater increase in H-D exchange in the α1-helix. After conditional ligand hydrolysis, there was increased H-D exchange throughout the α1-helix, similar to the β-strands. This suggests that, like the underlying β-strands, the hydrogen bonds of the α1-helix of peptide-receptive MHC I molecules open and subsequently allow backbone amide protons to exchange more rapidly than in the conditional ligand-loaded (non-UV-exposed) state. Interestingly, after conditional ligand hydrolysis, there was a greater increase in the incorporation of deuterium into the four turns of the α1-helix encoded by residues 68–80 (which spans from the center and extends toward the last turn of the α1-helix at the end of the groove that binds the peptide C terminus) than there was for the first five turns of the helix that are encoded by residues 57–71 (which start from the opposite end of the groove and extend to the center of the helix) (compare Fig. 6, *d* and *e*, *blue lines*). As observed for β-strands of the α1 domain, there was faster and, at least with these deuterium labeling periods, a greater increase in deuterium uptake for those polypeptides derived from the BF2*15:01 α1-helix than there was for the equivalent polypeptides derived from BF2*19:01.

The last region of the α1 domain was covered by polypeptide 81–90, which constitutes the last turn of the α1-helix and a six-residue loop leading to the α2 domain-encoded β-strand 5 (Fig. 6*f*). Within this loop is the asparagine residue (Asn-85, equivalent to Asn-86 in mammals), whose monoglucosylated *N*-linked glycan calreticulin binds. Mutations of this residue impair association with calreticulin and the other members of the PLC, and they negatively influence peptide binding (37). For polypeptide 81–90, even in the conditional ligand-loaded state, there was a substantial and rapid increase in the uptake of deuterium, with the majority of deuterium incorporated during the shortest labeling period, with little further increase in uptake over the longer labeling periods or following conditional ligand hydrolysis.

Collectively, this analysis of the α1 domain first suggests that some of the hydrogen bonds of the α1-helix and the connecting β-turns of conditional ligand-loaded MHC I molecules open and allow backbone amide protons to exchange more rapidly than those of the β-strands forming part of the floor of the peptide-binding groove. Second, in the peptide-receptive state, some of the hydrogen bonds of the β-strands and the α1-helix open and allow backbone amide protons to exchange at an even faster rate than in the conditional ligand-loaded state. Third, there is greater incorporation of deuterium toward the second half of the α1-helix (toward the end of the groove that binds the peptide C terminus) and the loop leading to the α2 domain than in the first part of the α1-helix. This shows that, at least as measured by HDX-MS, there is greater conformational plasticity in the α1 domain toward the peptide C terminus as suggested previously (20, 27, 28, 40, 49, 50). Fourth, there is faster and, at least with these deuterium-labeling periods, a greater increase in H-D exchange in polypeptides derived from β-strands and the α-helix of the BF2*15:01 α1 domain than is apparent for BF2*19:01.

### α2 domain-encoded β-strands

The α2 domain forms the second half of the peptide-binding groove, and encodes four β-strands (strands 5–8), their connecting β-turns, and an α-helix, which runs anti-parallel to the α1-helix and includes three sub-helices (Fig. 3). As in the α1 domain, the four β-strands form part of the floor of the peptide-binding groove, with these-strands situated toward the end of the groove that binds the peptide C terminus.

**Figure 7.**
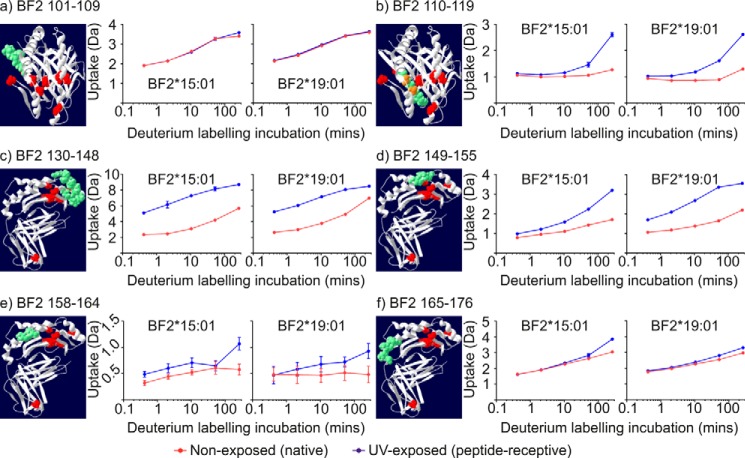
**Deuterium uptake charts for polypeptides derived from the α2 domains.** *a–f*, deuterium uptake charts for the indicated polypeptides derived from the α2 domains are presented as in Fig. 6.

Polypeptide 101–109, which constitutes the last two residues of β-strand 5, the first four residues of β-strand 6, and three residues connecting the β-hairpin turn (Fig. 7*a*), produced a similar exchange profile to that of polypeptides derived from β-turns located in the α1 domain (Fig. 6*c*). For residues 101–109, H-D exchange continued throughout the labeling period, and it was not affected by UV exposure. In contrast, the centrally located region of residues 110–119 (Fig. 7*b*), which constitutes the majority of β-strand 6, a single residue β-hairpin turn, and the first two residues of β-strand 7, showed negligible uptake of deuterium in the conditional ligand-loaded state. However, conditional ligand hydrolysis resulted in increased H-D exchange in both allotypes, similar to the β-strands of the α1 domain. This suggests that in contrast to β-strands on the periphery of the floor of the peptide-binding groove (as exemplified by polypeptides such as 101–109), conditional ligand hydrolysis disrupted hydrogen bonding in centrally located β-strands allowing the backbone amide hydrogens to participate in H-D exchange much faster than in the conditional ligand-loaded state (as is evident for polypeptides such as 110–119).

### α2–1 sub-helix and the underlying β-strand 8

Numerous polypeptides derived from β-strand 8, the α2–1 sub-helix, the β-turn connecting these regions, and the start of the α2–2 sub-helix were detected. These polypeptides are of particular interest as they include residues equivalent to the loop formed by residues 128–136 of mammalian MHC I molecules (chicken residues 125–133 (51)), to which the N-terminal domain of tapasin is thought to bind (12, 34, 37, 52). Mutations made within this loop region generally impair or completely ablate association with tapasin and the PLC, and consequently negatively influence MHC I peptide loading. As exemplified by polypeptide 130–148 (Fig. 7*c*), in the conditional ligand-loaded state the uptake of deuterium increased throughout the labeling period. Hydrolysis of the conditional ligand increased the speed of H-D exchange, which was apparent even after the shortest incubation in deuterium. In keeping with the results of the β-strands and α-helix of the α1 domain, there was faster and, at least during these deuterium labeling periods, greater increase in the uptake of deuterium in polypeptides derived from the BF2*15:01 allotype than there was for equivalent polypeptides derived from BF2*19:01.

### α2–2 sub-helix

The H-D exchange profile of polypeptide 149–155, which maps to the α2–2 sub-helix of both allotypes, is shown in Fig. 7*d*. Similar to the α2–1 sub-helix and underlying β-strand 8, UV exposure brought about an increase in H-D exchange for both allotypes, which was apparent even after the shortest incubation period. Notably, however, this increase was faster and, at least with these deuterium labeling periods, greater for polypeptides derived from peptide-receptive BF2*19:01 molecules than for equivalent polypeptides derived from peptide-receptive BF2*15:01 molecules. This allotype-specific difference in H-D exchange was also observed following conditional ligand hydrolysis with polypeptides, including 110–119, which constitute most of β-strand 6 and the start of β-strand 7 and are in close proximity beneath the α2–2 sub-helix (Fig. 7*b*, *e.g.* compare deuterium uptake for incubation times greater than 2 min). Notably, the α2–2 sub-helix and the underlying β-strands were the only examples where conditional ligand hydrolysis resulted in faster and greater exchange in the BF2*19:01 allotype than there was in the BF2*15:01 allotype. Furthermore, amino acid residues 149–155 are one of the regions where greater H-D exchange was observed in the BF2*19:01 allotype in the conditional ligand-loaded state than in BF2*15:01 (polypeptide 131–155 is shown in Fig. 5*f*, see supplemental Fig. S2 for polypeptide 149–155).

### α2–3 sub-helix

Two consecutive polypeptides covered the α2–3 sub-helix, and both showed much lower levels of deuterium uptake in the conditional ligand-loaded state than the α2–1 and α2–2 sub-helices, especially in the first part of the α2–3 sub-helix (Fig. 7, *e*, polypeptide 158–164, and *f*, polypeptide 165–176). After conditional ligand hydrolysis, there was only a slight increase in H-D exchange, which was most apparent with the longest deuterium labeling period. This suggests that there is slower opening of backbone hydrogen bonds, and subsequently H-D exchange, and that the local environment of the backbone amide hydrogens of the α2–3 sub-helix is less affected by hydrolysis of the conditional ligand than the other α2 sub-helices, as suggested previously (20, 27, 40, 49, 50). As observed with polypeptides derived from the α-helix and β-strands of the α1 domain, as well as in the α2–1 sub-helix (but not the α2–2 sub-helix), there was faster and, at least during these deuterium-labeling periods, greater H-D exchange occurring in polypeptides derived from the BF2*15:01 α2–3 sub-helix than was apparent for their BF2*19:01 equivalents.

### MHC I α3 and β_2_m domains demonstrate allotype-specific long range communication of dynamic events following conditional ligand hydrolysis

**Figure 8.**
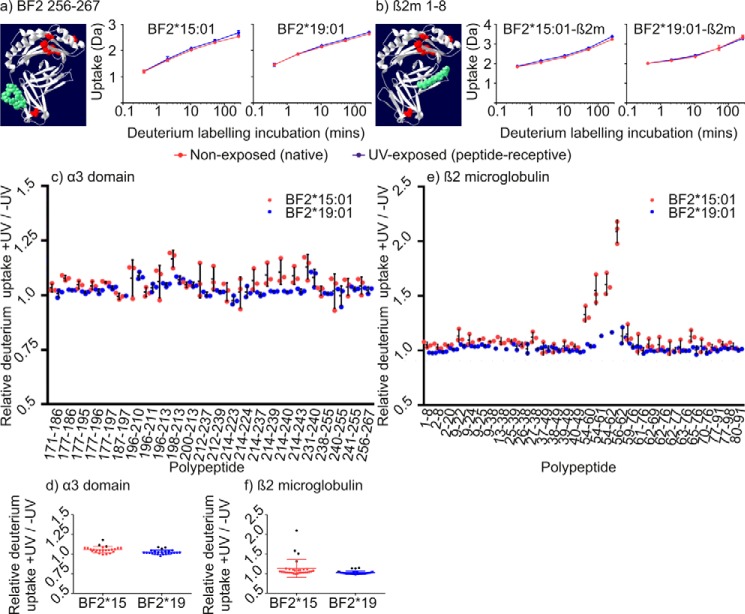
**Deuterium uptake in the α3 and β_2_m domains.** *a* and *b* show deuterium uptake charts for the indicated polypeptides derived from the α3 (*a*) or β_2_m (*b*) domains, presented as in Fig. 6. *c* shows for each polypeptide derived from the α3 domains of the two allotypes the relative difference that conditional ligand hydrolysis makes to deuterium uptake normalized to the uptake of non-exposed samples after incubation in deuterated buffer for 4.34 h (*i.e.* for each polypeptide the increase in deuterium uptake after conditional ligand hydrolysis was divided by the increase in deuterium uptake of the non-exposed sample after labeling for 4.34 h). For each polypeptide, *dots* denote the normalized uptake of deuterium for each of the replicate samples; *horizontal bars* denote the mean uptake for all replicates; and *vertical lines* denote the standard deviation. *d* shows the normalized deuterium uptake of individual polypeptides as *dots*, colored according to allotype, with the standard deviations depicted by *vertical lines* edged with *short horizontal bars*, and the mean average normalized uptake of all polypeptides as a *long horizontal bar*. Polypeptides discussed in the text are denoted by *black dots*. Conditional ligand hydrolysis resulted in a significant increase in the uptake of deuterium into the polypeptides derived from the α3 domain (paired two tailed *t* tests, BF2*15:01 comparison of ± UV, *p* = <0.0001; BF2*19:01 comparison of ± UV, *p* = 0.0002). *e* and *f* show polypeptides derived from β_2_m as presented as in *c* and *d*.

The MHC I peptide-binding domain sits atop two membrane-proximal immunoglobulin-like domains: the MHC I-encoded α3 domain, which contains a single polymorphic residue at position 220 in β-strand 4, and the monomorphic and non-covalently associated β_2_m subunit. In comparison with sub-regions of the peptide-binding domain, conditional ligand hydrolysis resulted in smaller increases in H-D exchange in the α3 and β_2_m domains of both MHC I allotypes (Figs. 2, *a* and *c,* and 4). As exemplified by BF2 polypeptide 256–267 (Fig. 8*a*) and β_2_m polypeptide 1–8 (Fig. 8*b*), UV exposure had little or no effect on the incorporation of deuterium into most of the polypeptides derived from these domains (see supplemental Figs. S4 and S5 for deuterium uptake charts for all polypeptides). The comparatively modest increases in H-D exchange that follow conditional ligand hydrolysis suggest that the opening rates of backbone hydrogen bonds of these membrane-proximal domains, and their subsequent participation in H-D exchange, is less affected by conditional ligand hydrolysis than in the peptide-binding domain. This is consistent with these membrane-proximal domains not directly interacting with the conditional ligand. However, despite the comparatively small scale of the increases in deuterium uptake that conditional ligand hydrolysis caused in any of the 24 polypeptides derived from the α3 domain (Fig. 8*c*), these increases were significant, varied between allotypes, and were greatest in particular sub-regions of the α3 domain.

**Figure 9.**
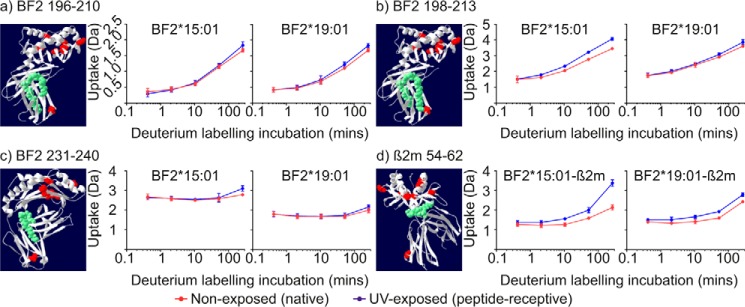
**Deuterium uptake charts for polypeptides derived from the α3 and β_2_m domains.** *a–d*, deuterium uptake charts for the indicated polypeptides derived from the α3 or β_2_m domains of the two allotypes are presented as in Fig. 6.

We found that polypeptides 196–210, 198–213, and 231–240 exhibited the largest increases in the incorporation of deuterium into the α3 domains of both allotypes after conditional ligand hydrolysis (Fig. 8*d*, with these polypeptides denoted by *black dots*, and Fig. 9, *a–c*). Residues 196–213 constitute most of β-strands 2 and 3, as well as their connecting residues, whereas residues 231–240 cover most of β-strand 6, half of β-strand 7, and a three-residue connecting β-hairpin turn. Interestingly, these regions are located beneath the peptide-binding domain and are situated toward the end that binds the peptide C terminus. As with the peptide-binding domains, differences between the two allotypes were apparent, albeit more subtle, with generally greater incorporation of deuterium into polypeptides derived from the α3 domain of the BF2*15:01 allotype.

Comparison of deuterium uptake for the 30 β_2_m-derived polypeptides detected for both allotypes clearly identified one region where conditional ligand hydrolysis resulted in a significant increase in H-D exchange (Fig. 8, *e* and *f*, where polypeptides 54–60, 54–61, 54–62, and 56–62 are denoted by *black dots*). Residues 54–62 constitute β-strand 6, the start of the β-strand 7, and their connecting β-hairpin turn (Fig. 9*d*). Notably, as with BF2 polypeptides 196–213 and 231–240, β_2_m residues 54–62 are located immediately beneath, and interact with, the peptide-binding domain. As before, conditional ligand hydrolysis resulted in greater uptake of deuterium into this portion of the β_2_m molecule associated with the BF2*15:01 allotype.

**Figure 10.**
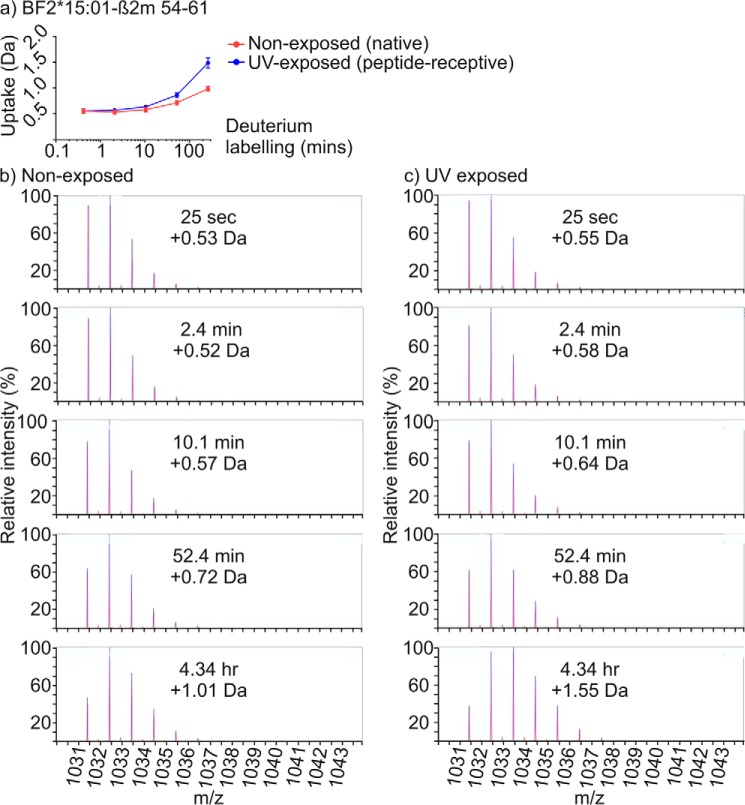
**Comparison of mass spectra of β_2_m polypeptide 54–61 with or without prior UV exposure.** *a,* deuterium uptake chart for polypeptide 54–61 derived from the β_2_m molecule associated with the BF2*15:01 complex as presented as in Fig. 6. *b,* individual mass spectra of the +1 ion of polypeptide 54–61 (*m/z* 1031 [M + H]^+^) derived from β_2_m associated with the BF2*15:01 complex after incubation in deuterium for the indicated length of time without prior exposure to UV light. The increase in mass (relative to the undeuterated polypeptide) is indicated. One of the three replicates is shown. The peaks that were used for the uptake calculations (shown in Fig. 8*a*) are marked with *blue bars. c,* as for *b*, but the samples were UV-exposed prior to the incubation in deuterium.

It is possible that the small increases in deuterium uptake that we observed in sub-regions of the α3 and β_2_m domains might result from the dissociation of a proportion of HC–β_2_m complexes into free HC and β_2_m molecules, with greater H-D exchange occurring in the separate proteins than in HC–β_2_m complexes. However, three lines of evidence suggest that this is not the case. First, Fig. 10 shows that for the 1+ ion of β_2_m polypeptide 54–61 associated with the BF2*15:01 complex, which has one of the largest increases in deuterium uptake following conditional ligand hydrolysis (Figs. 8*e* and 10*a*), there is a single population of peptides observed in the mass spectra irrespective of UV exposure, and not a bimodal distribution that might result from dissociation of a proportion of HC–β_2_m complexes (Fig. 10, *b* and *c*). Second, excess peptide completely reverses the UV induced changes in H-D exchange profiles (Fig. 2). Third, whereas the increases in deuterium uptake that occur in the membrane-proximal domains are greater for BF2*15:01 following conditional ligand hydrolysis (Figs. 4, 8 and 9) it is peptide-receptive BF2*19:01 molecules that lose the ability to bind peptide faster, and whose HC–β_2_m complexes might be expected to dissociate more rapidly (Fig. 1). Instead, the conditional ligand hydrolysis-dependent changes in deuterium uptake that occur in these membrane-proximal regions are likely to reflect long-range communication between the peptide-binding domain and the α3 and β_2_m domains, as suggested previously from molecular dynamics simulations (20, 27, 28, 40). Our findings are also consistent with the reduced incorporation of deuterium into portions of the HLA-A*02:01 α3 domain following ligation of the peptide-binding domain with T cell receptor (53). By extension, the observation that deuterium uptake was greater in the α3 and β_2_m domains of the BF2*15:01 allotype suggest that this long-range communication might differ between allotypes.

## Discussion

We have directly compared the dynamic properties, as measured by HDX-MS, of two MHC I allotypes in their conditional ligand-loaded and in their peptide-receptive states. Our comprehensive analysis of 102 polypeptides, whose location within the protein sequence was common to both allotypes, showed that for the majority of polypeptides derived from the conditional ligand-loaded proteins there was no significant difference in deuterium uptake between the two allotypes; however, conditional ligand hydrolysis differentially influenced H-D exchange in these molecules. HDX-MS experiments involving MHC I proteins had previously been limited to peptide-loaded complexes. For example, the H-D exchange profile of peptide-HLA-A*02:01 complexes was shown to be influenced by the sequence of the bound peptide (54). Additionally, ligation of a peptide-HLA-A*02:01 complex with its cognate T cell receptor decreased H-D exchange in both molecules, suggesting ligation rigidified both proteins (53). Similarly, another study found there was greater H-D exchange in free β_2_m compared with peptide-MHC bound β_2_m (55).

We observed that conditional ligand hydrolysis increased the exchange of backbone amide hydrogens of the peptide-binding domain to a greater extent than in the membrane-proximal α3 and β_2_m domains. This demonstrates that hydrogen bonds of the peptide-binding domain open and allow backbone amide protons to exchange more rapidly following hydrolysis of the conditional ligand, and the dissociation of the resultant peptide fragments, than in the membrane-proximal domains. Importantly, the increases in H-D exchange we observed were completely reversed by the addition of peptide after UV exposure, confirming that the H-D exchange profile of UV exposed BF2*19:01 molecules was indicative of peptide-receptive molecules. The fact that the H-D exchange profile of UV-exposed and peptide-supplemented BF2*19:01 molecules resembled that of BF2*19:01 molecules that were not UV-exposed demonstrates that any fragments of the hydrolyzed conditional ligand that might remain within the groove must be bound with exceptionally low affinity, such that they are rapidly and comprehensively exchanged with the KRLIGKRY peptide. Notably, the increased uptake of deuterium that resulted following dissociation of the peptide cargo did not occur uniformly throughout the peptide-binding domain. For example, the increase in H-D exchange was largest and fastest toward the end of the peptide-binding groove that binds the peptide C terminus and which includes the α2–1 sub-helix, underlying β-strand 8 and their connecting residues. This demonstrates that there is greater conformational plasticity, as measured by H-D exchange, around the peptide C terminus in the peptide-receptive MHC I peptide-binding groove than around the peptide N terminus. Interestingly, the conformational plasticity of the MHC I peptide-binding groove region around the peptide C terminus has previously been predicted to be influenced by either the absence of peptide or to differ between allotypes (20, 27, 40, 50, 56). Furthermore, we observed that even in the conditional ligand-loaded state, substantial H-D exchange occurred in these sub-regions, as with the α1-helix. This suggests the interactions between these HC sub-regions and the conditional ligand are dynamic.

Our comparison of H-D exchange in the two MHC I allotypes showed that the increases in deuterium uptake that resulted from conditional ligand hydrolysis occurred in similar locations for both allotypes (Fig. 4). However, for many polypeptides the rates of deuterium uptake differed between the allotypes following UV-induced hydrolysis of the conditional ligand. Although it is possible that the eight polymorphic differences between the two allotypes might influence the efficiency at which the conditional ligand is hydrolyzed, we do not consider this likely to account for the allotype-specific differences that we have observed in H-D exchange. For example, if the efficiency of conditional ligand hydrolysis were to differ significantly between the allotypes, it might be anticipated that the comparison of UV-exposed and non-exposed samples would differ markedly between the allotypes, yet we observed both allotypes share a common overall pattern of increased H-D exchange after UV-induced peptide hydrolysis.

We found that there was a general trend of faster and greater increases in H-D exchange occurring in the BF2*15:01 allotype after conditional ligand hydrolysis. This was apparent for polypeptides derived from the α-helix and β-strands of the α1 domain, the α2–1 and α2–3 sub-helices, and several underlying β-strands of the α2 domain. Interestingly, this was also evident with sub-regions of the membrane-proximal α3 domain and β_2_m molecule, where in the peptide-receptive state H-D exchange increased for several polypeptides derived from the top portions of the α3 and β_2_m domains to a greater extent for BF2*15:01 than was apparent for BF2*19:01. As these regions are in closest proximity to the underside of the peptide-binding groove, they may indicate pathways by which dynamic changes brought about by the hydrolysis of the conditional ligand are communicated from the peptide-binding domain to the membrane-proximal domains. In contrast, however, there was faster and greater incorporation of deuterium into the α2–2 sub-helix and polypeptides derived from two underlying β-strands of the BF2*19:01 molecule after conditional ligand hydrolysis. Cumulatively, this suggests that the dynamic profiles of the two allotypes differed in their peptide-receptive states.

We have previously found that BF2*15:01 has a greater intrinsic ability to select and assemble with high-affinity peptides, and it receives less enhancement from tapasin than BF2*19:01 (19, 20). We have also shown that BF2*15:01 is able to explore a greater conformational space and adopts more alternative conformations in the absence of peptide than BF2*19:01 in molecular dynamic simulations and that the peptide-selecting function of MHC I molecules correlates with the plasticity of peptide-empty MHC I molecules in these experiments (20, 27, 28, 40). Our observation that the two allotypes have different dynamic profiles in their peptide-receptive states, as measured by HDX-MS, complements these findings and is consistent with the notion that conformational plasticity underpins the peptide-selecting function of MHC I molecules.

The MHC I loading cofactor tapasin contacts MHC I via two sites of interaction (33–38). The N-terminal domain of tapasin is thought to bind around the MHC I α2–1 sub-helix and underlying β-strands, where conditional ligand hydrolysis caused the largest and fastest increases in H-D exchange in comparison with the non-exposed state. The membrane-proximal domain of tapasin is thought to bind to the MHC I α3 domain where, in contrast, H-D exchange occurred extremely rapidly and, at least as far as we were able to determine, was unaffected by conditional ligand hydrolysis. We have suggested that tapasin uses these dual interaction surfaces to modulate MHC I plasticity and thereby the peptide selecting function (27, 28). MHC I allotypes such as BF2*19:01, which molecular dynamic simulations suggest are intrinsically inefficient at transiting between alternative conformations in the absence of peptide and which predominantly occupy a single energy minimum (20, 40), may require tapasin to catalyze the adoption of different intermediate states and in so doing select peptides efficiently. Conversely, allotypes such as BF2*15:01 transiently occupy multiple and distinct energy minima during molecular dynamic simulations conducted in the absence of peptide. BF2*15:01 molecules are therefore likely to be intrinsically more efficient at discriminating between peptides because they transition more rapidly between intermediate states without the assistance of tapasin (20, 27, 28, 40).

In conclusion, our comparison of MHC I molecules in their conditional ligand-loaded and peptide-receptive states has shown that MHC I molecules are dynamic proteins, at least as measured by HDX-MS. Consistent with the notion that an intermediate MHC I state is important for efficient peptide selection (27), we observed that the dynamic properties of MHC I molecules change depending upon the occupancy of the peptide-binding groove. Our comparison of two MHC I allotypes has shown that they differ in their H-D exchange profiles in the peptide-receptive state, which correlates with previous findings that these allotypes differ in peptide-selecting function (19, 20) and in the adoption of alternative energy minima in molecular dynamic simulations in the peptide-empty state (20, 40). Collectively, these studies suggest that the ability of MHC I molecules to explore alternative states underpins their ability to sample peptides efficiently (20, 27, 28, 40).

## Experimental procedures

### Production of peptide-loaded and peptide-receptive BF2 proteins

BF2*15:01 or BF2*19:01 HC proteins were produced and refolded with chicken β_2_m and the UV-labile peptide KRLIGjRY (Peptide Synthetics; j represents 3-amino-3-(2-nitro)phenylpropionic acid) as described in Ref. 19. Peptide-receptive BF2 complexes were generated by exposing BF2-KRLIGjRY complexes to 366 nm light for 20 min at 4 ºC or on ice (UV-exposed).

### Fluorescence polarization experiments

A final concentration of 1 μm BF2*15:01-KRLIGjRY or BF2*19:01-KRLIGjRY complexes, prepared in PBS supplemented with 0.5 mg/ml bovine γ-globulin (Sigma), were UV-exposed before being incubated at 25 ºC. At the indicated time points, samples were taken and incubated with 229 nm KRLIGK*RY (GL Biochem, K* denotes TAMRA-labeled lysine) for 1 h at 25 ºC. Fluorescent polarization measurements of 60-μl samples were taken using an I3x (Molecular Devices) with rhodamine detection cartridge. Binding of TAMRA-labeled peptides is reported in millipolarization units (mP) and is obtained from the equation mP = 1000 × (*S* − *G* × *P*)/(*S* + *G* × *P*), where *S* and *P* are background-subtracted fluorescence count rates (*S* = polarization emission filter is parallel to the excitation filter; *P* = polarization emission filter is perpendicular to the excitation filter; and *G* (grating) is an instrument and assay-dependent factor).

### Hydrogen–deuterium exchange mass spectrometry

#### 

##### Sample preparation for the experiment shown in Fig. 2

Initial samples of 20 μm BF2*19:01-KRLIGjRY were prepared using equilibration buffer (10 mm potassium phosphate, pH 7.0), which were then either 1) kept on ice without exposure to UV light; 2) UV-exposed for 20 min on ice; or 3) UV-exposed and then supplemented with 10-fold molar excess of KRLIGKRY (GL Biochem) peptide immediately after the UV exposure.

##### Sample preparation for the experiment shown in Figs. 4–10

Initial samples of 20 μm BF2*15:01-KRLIGjRY complexes or BF2*19:01-KRLIGjRY complexes were prepared using equilibration buffer, which were then either 1) kept on ice without exposure to UV light, or 2) UV-exposed for 20 min on ice.

##### Hydrogen–deuterium exchange

An automated system (HDX PAL operated by Chronos 4.0 software, Leap Technologies) was utilized to schedule replicate injections for numerous deuterium-exchange incubation periods. Samples were diluted into either equilibration buffer or deuterium oxide buffer (10 mm potassium phosphate, pD 7.0), before the exchange reaction was quenched. Deuterium labeling conditions are as follows: 3 μl of samples of 20 μm UV-exposed or non-exposed BF2*15:01 or BF2*19:01 complexes were diluted 20-fold into either equilibration buffer or deuterium oxide buffer. The proteins were incubated for 25 s, 10.4 min, or 4.34 h for the experiment shown in Fig. 2, or for 25 s, 2.1, 10.4, or 52.1 min, or 4.34 h for the experiment shown in Figs. 4–10, prior to quenching in an equal volume of 100 mm potassium phosphate, 2.0 m guanidine hydrochloride, 250 mm tris(2-carboxyethyl)phosphine, H_2_O, pH 2.30. Quenched proteins (25 pmol) were injected into a temperature-controlled chromatography chamber (HDX Manager, Waters) in which integrated on-line pepsin digestion was performed (Enzymate column, Waters) at ∼7000 p.s.i., 20 °C, followed by chromatographic separation at 0 °C (Acquity M-class UPLC, Waters). Eluted peptides were detected via an MS^E^ acquisition method using a Synapt G2-Si mass spectrometer (Waters). Data processing was performed using ProteinLynx Global Server version 3.0.2 and DynamX version 3.0 (Waters) using the following parameters for compiling the library of peptic peptides: non-deuterated peptide precursor accurate mass tolerance 4 ppm; minimum average of 0.3 fragment ions per residue; reproducibility, 4 of 5 replicates. All deuterated data were acquired in triplicate for every time point and state.

We found that the kinetics of H-D exchange were highly reproducible between experiments; supplemental Fig. S6 compares the uptake of deuterium for all polypeptides derived from BF2*19:01 in the three-state experiment (results shown in Fig. 2), which were also observed for both BF2 allotypes in the two-state experiment (results shown in Figs. 4–10).

We observed that the uptake of deuterium varied between polypeptides (supplemental Figs. S2–S5), but generally increased with increasing incubation time in deuterated buffer. We found that two of the shorter polypeptides, BF2 polypeptide 50–56 (7 amino acids) and β_2_m polypeptide 2–8 (7 amino acids), showed some of the largest increases, around 64 or 60%, respectively, of their maximum theoretical uptake, after 4.34-h incubation in deuterated buffer.

A paired two-tailed *t* test was used to determine whether the increases in deuterium uptake that occurred in the α3 domain after conditional ligand hydrolysis were significant (BF2*15:01 comparison of ± UV *p* = <0.0001, BF2*19:01 comparison of ± UV *p* = 0.0002).

## Author contributions

A. v. H., A. B., P. S., and T. E. conceived and coordinated the study. A. v. H. designed, performed and analyzed the experiment shown in Fig. 1. A. v. H. and M. A. designed and M. A. performed the experiments shown in Figs. 2 and 4–10. A. v. H., A. B., and M. A. analyzed the results of Figs. 2 and 4–10. A. v. H. wrote the article. A. v. H., M. A., A. B., J. M. W., P. S., and T. E. reviewed the results and critically revised the article for important intellectual content and approved the final version of the manuscript.

## Supplementary Material

Supplemental Data

## Footnotes

2 The abbreviations used are:

MHC I: major histocompatibility complex class I molecules
MHC: major histocompatibility complex
HC: heavy chain
β_2_m: β_2_-microglobulin
ER: endoplasmic reticulum
PLC: peptide-loading complex
HLA: human leukocyte antigen
HDX-MS: hydrogen–deuterium exchange mass-spectrometry
H-D: hydrogen–deuterium
mP: millipolarization unit
TAMRA: tetramethylrhodamine.
