# Supplementary material for: Direct evidence for conformational dynamics in major histocompatibility complex class I molecules
Source: J Biol Chem. 2017 Oct 11;292(49):20255–69. doi: 10.1074/jbc.M117.809624 (PMC5724011; doi:10.1074/jbc.M117.809624)
Supplement: Supplemental Data [file supp_292_49_20255__index.html]

Direct evidence for conformational dynamics in Major Histocompatibility Complex class I molecules — Direct evidence for conformational dynamics in major histocompatibility complex class I molecules — Evidence for conformational dynamics in MHC I allotypes — Supplemental Data 

# Direct evidence for conformational dynamics in major histocompatibility complex class I molecules

## Supplemental Data

- supplementary figures (.pdf, 12.8 MB) - PDF containing supplementary figures 1 to 6.
