# Supplementary material for: Direct evidence for conformational dynamics in major histocompatibility complex class I molecules
Source: J Biol Chem. 2017 Oct 11;292(49):20255–69. doi: 10.1074/jbc.M117.809624 (PMC5724011; doi:10.1074/jbc.M117.809624)
Supplement: Supplemental Data [file 10.1074_M117.809624_jbc.M117.809624-1.pdf]

Supplementary figure 1

a) BF2*19:01 HC: “non-exposed” vs. “exposed” vs. “exposed + peptide supplemented”

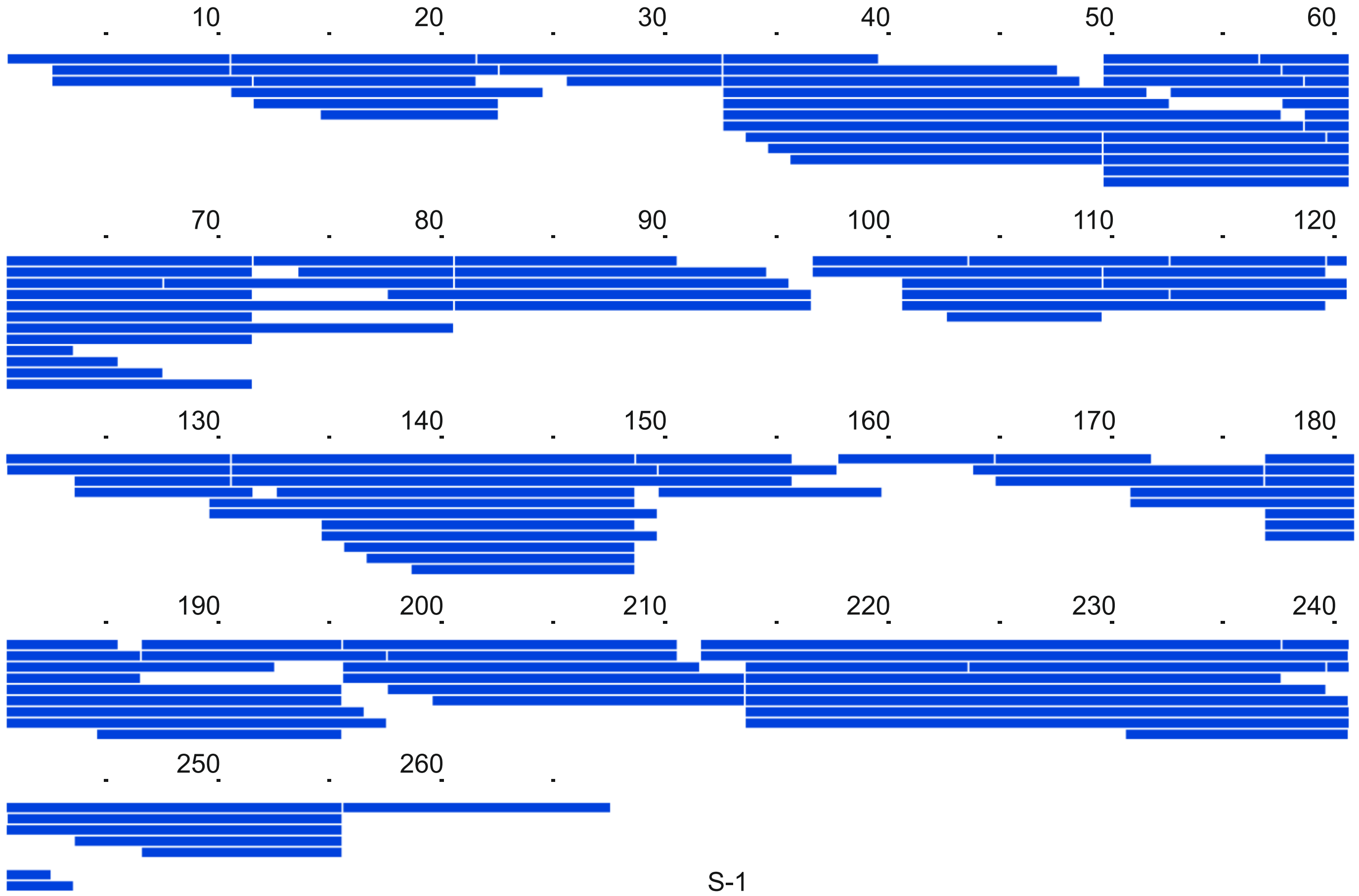

Supplementary figure 1

b) BF2*19:01 ß2m: “non-exposed” vs. “exposed” vs. “exposed + peptide supplemented”

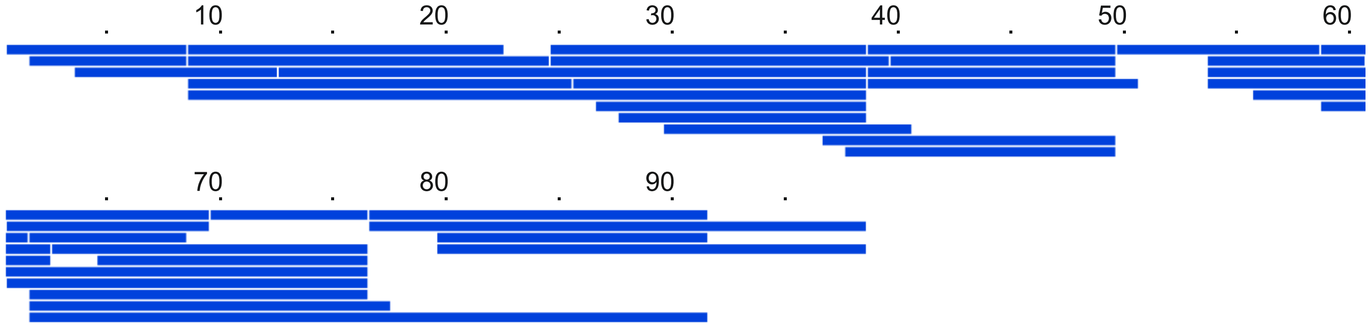

c) BF2*15:01 ß2m: “non-exposed” vs. “exposed”

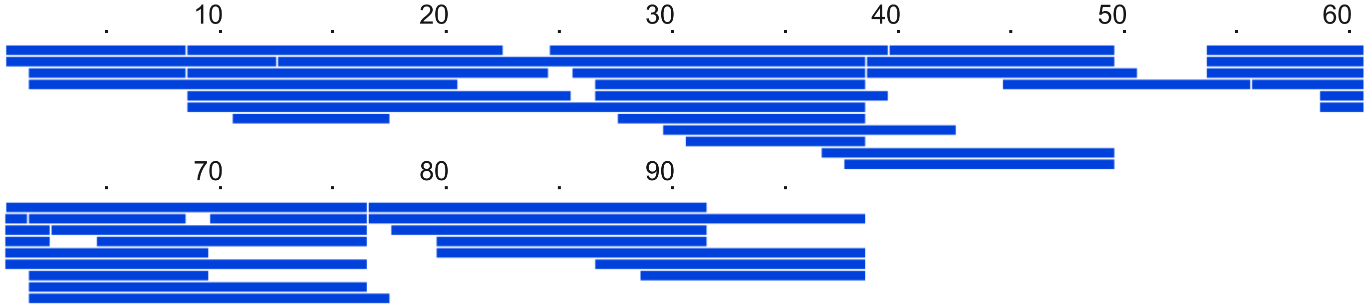

d) BF2*19:01 ß2m: “non-exposed” vs. “exposed”

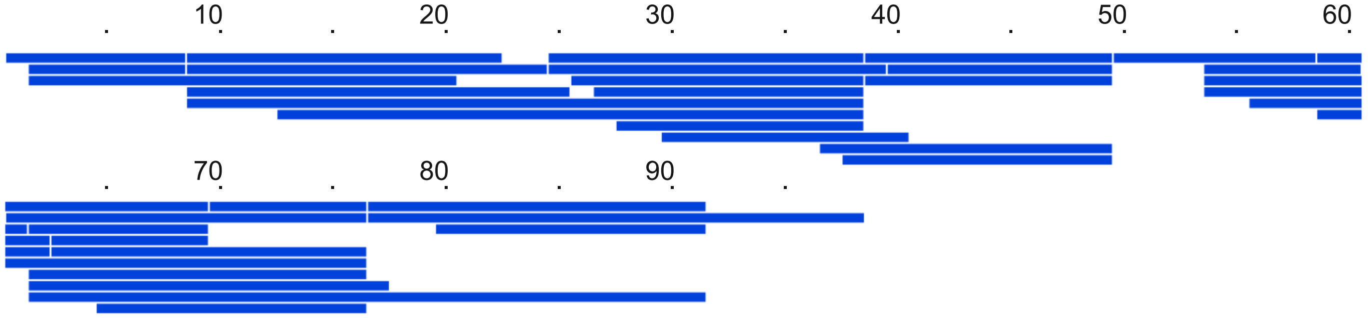

Supplementary figure 1

e) BF2*15:01 HC: “non-exposed” vs. “exposed”

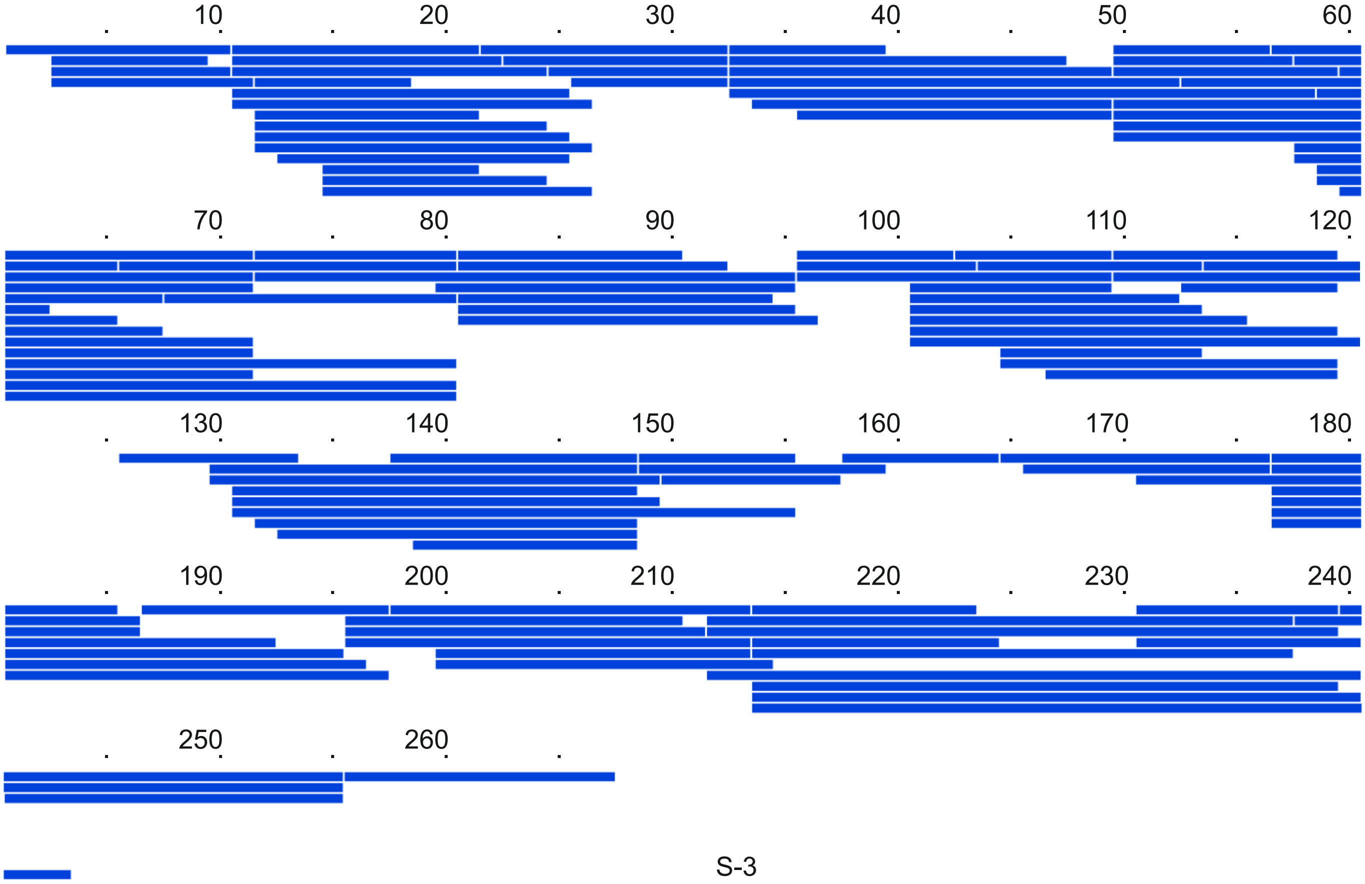

Supplementary figure 1

f) BF2*19:01 HC: “non-exposed” vs. “exposed”

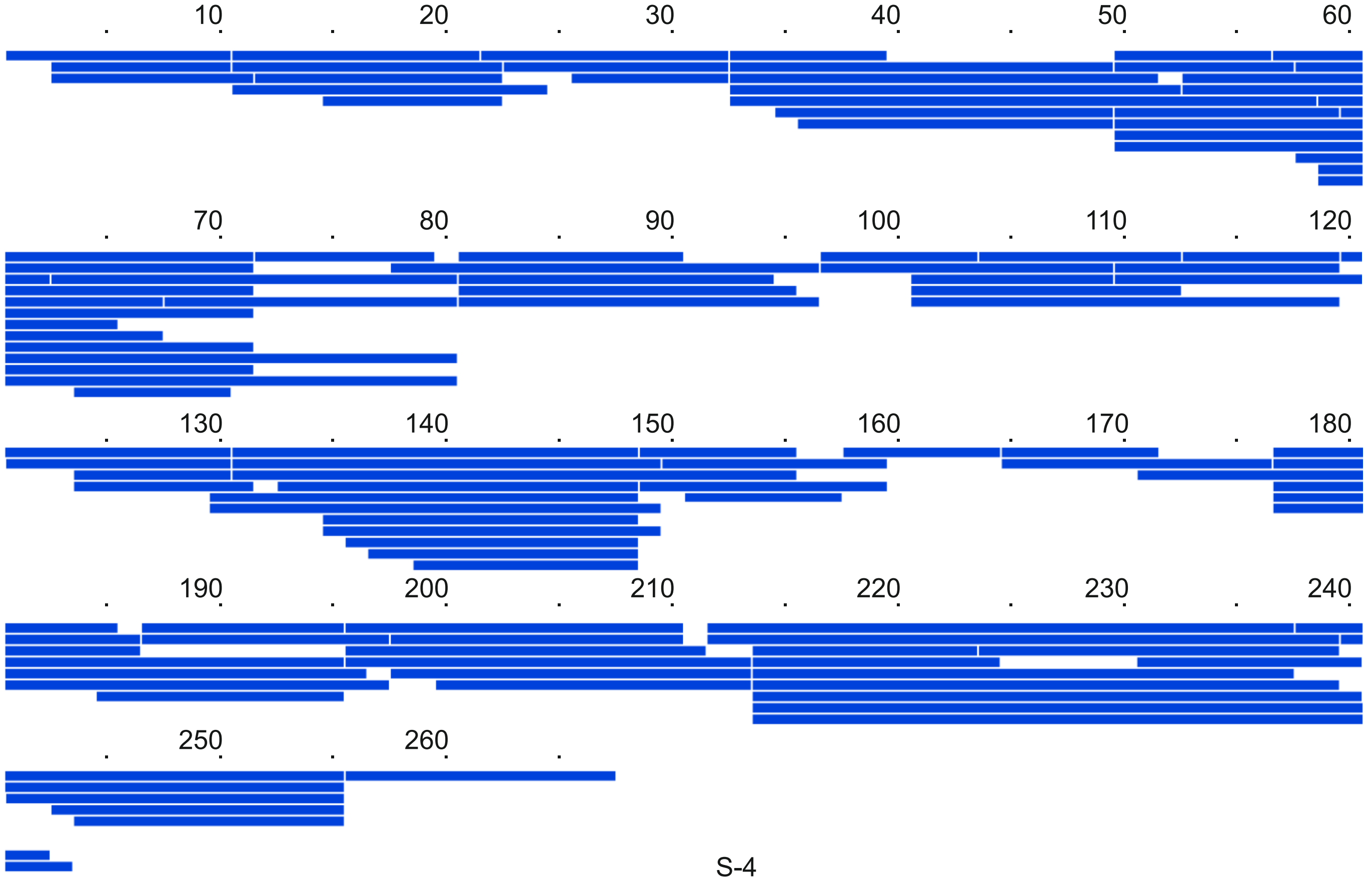

Supplementary figure 1 Polypeptide coverage maps.

The location of polypeptides derived from either A) BF2*19:01 HC or B) β_2m associated with the BF2*19:01 complex, where deuterium labelling occurred for 25 seconds, 10.4 minutes, or 4.34 hours before quenching (results shown in figure 2), in which non-exposed samples were compared with UV exposed samples, and also with UV exposed samples to which excess KRLIGKRY peptide had been added. Panels C and D show polypeptides derived from β_2m associated with the BF2*15:01 (C) or the BF2*19:01 (D) complexes, and panels E and F show polypeptides derived from BF2*15:01 HC (E) or BF2*19:01 HC (F), where deuterium labelling occurred for 25 seconds, 2.1, 10.4, 52.1 minutes, or 4.34 hours before quenching, in which non-exposed samples were compared with UV exposed samples (results shown in figures 4-10). The x axis denotes the protein sequence, with every fifth residue aligned with a period (.) and every tenth residue numbered.

Supplementary figure 2

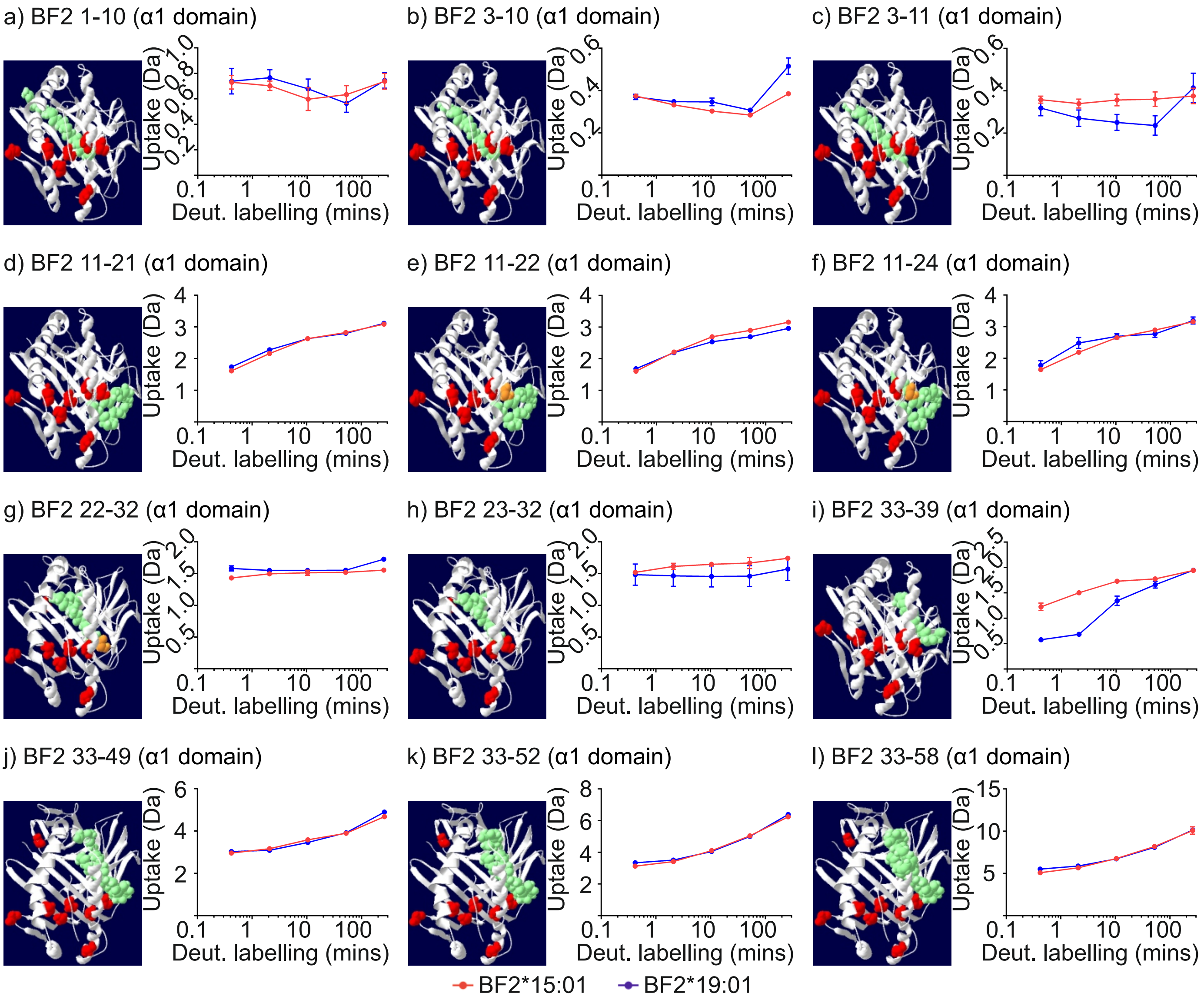

Supplementary figure 2

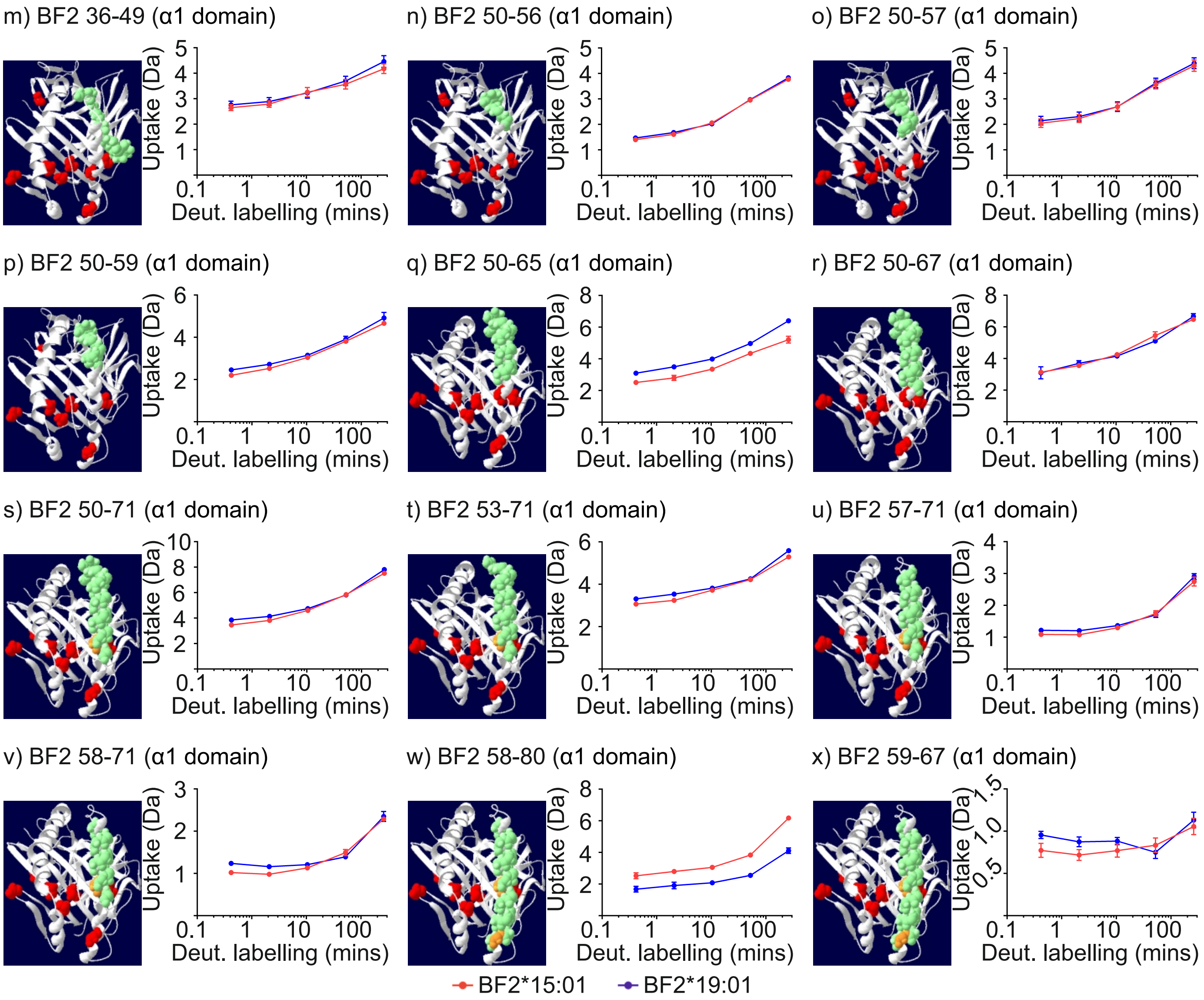

Supplementary figure 2

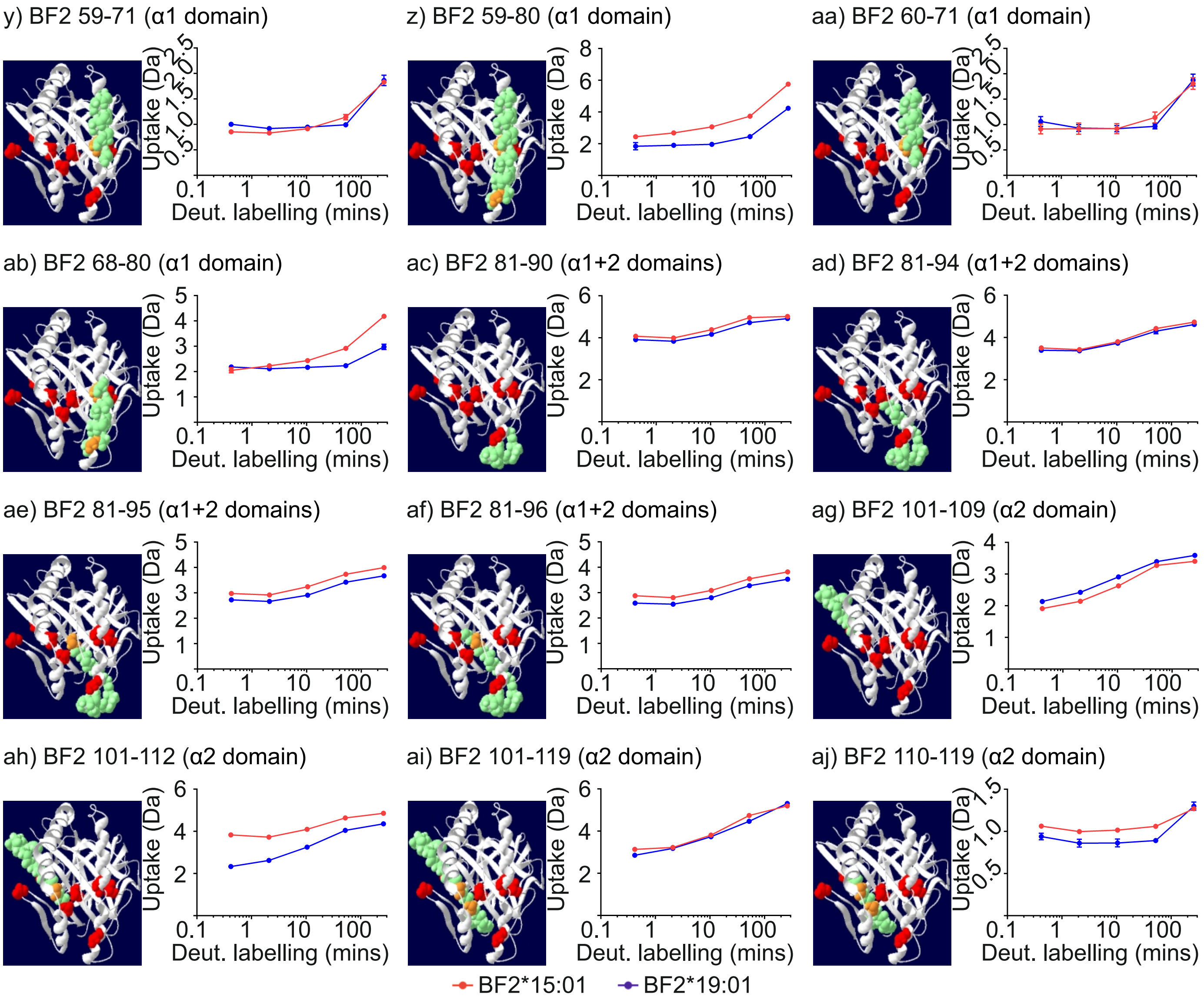

Supplementary figure 2

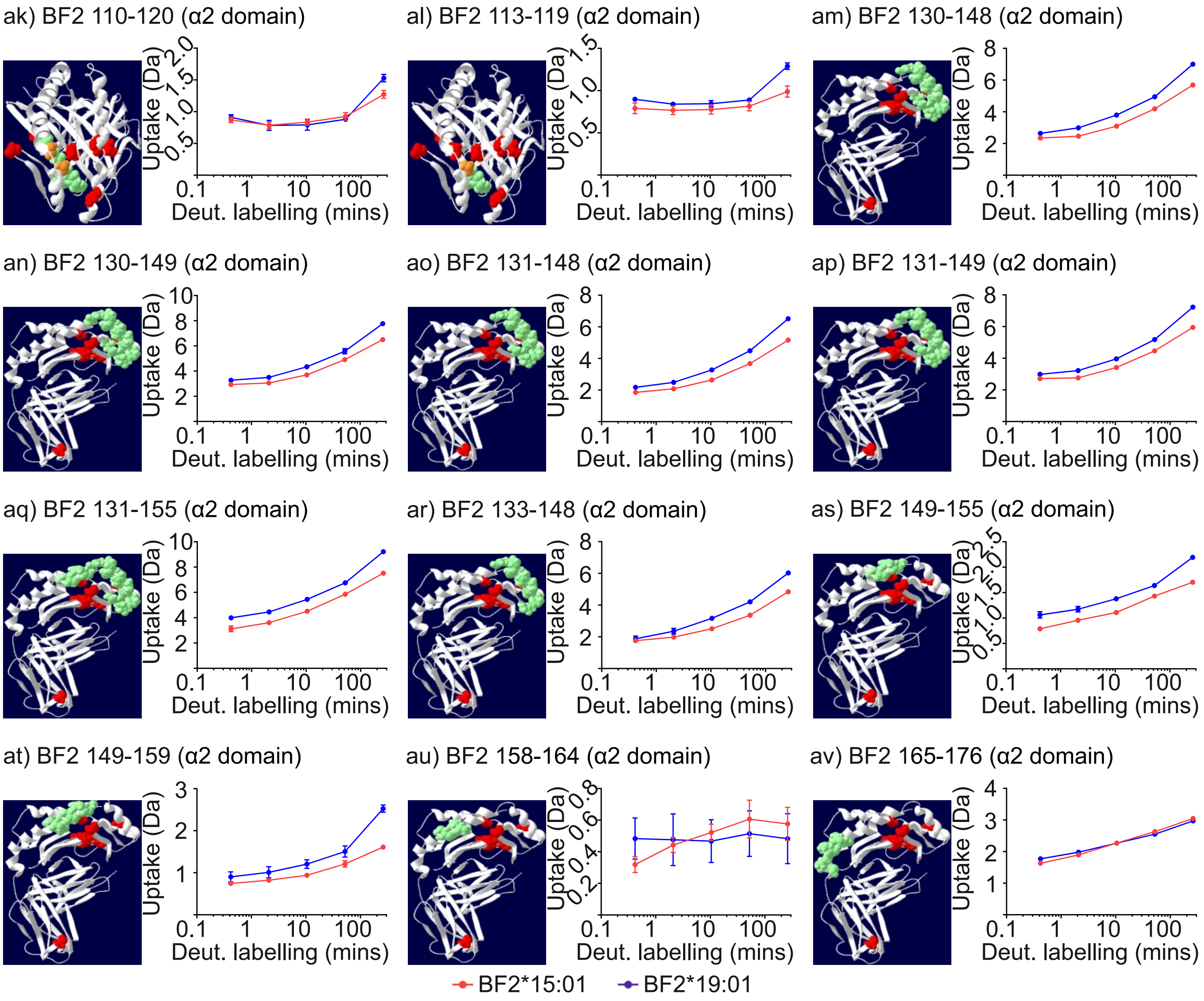

Supplementary figure 2

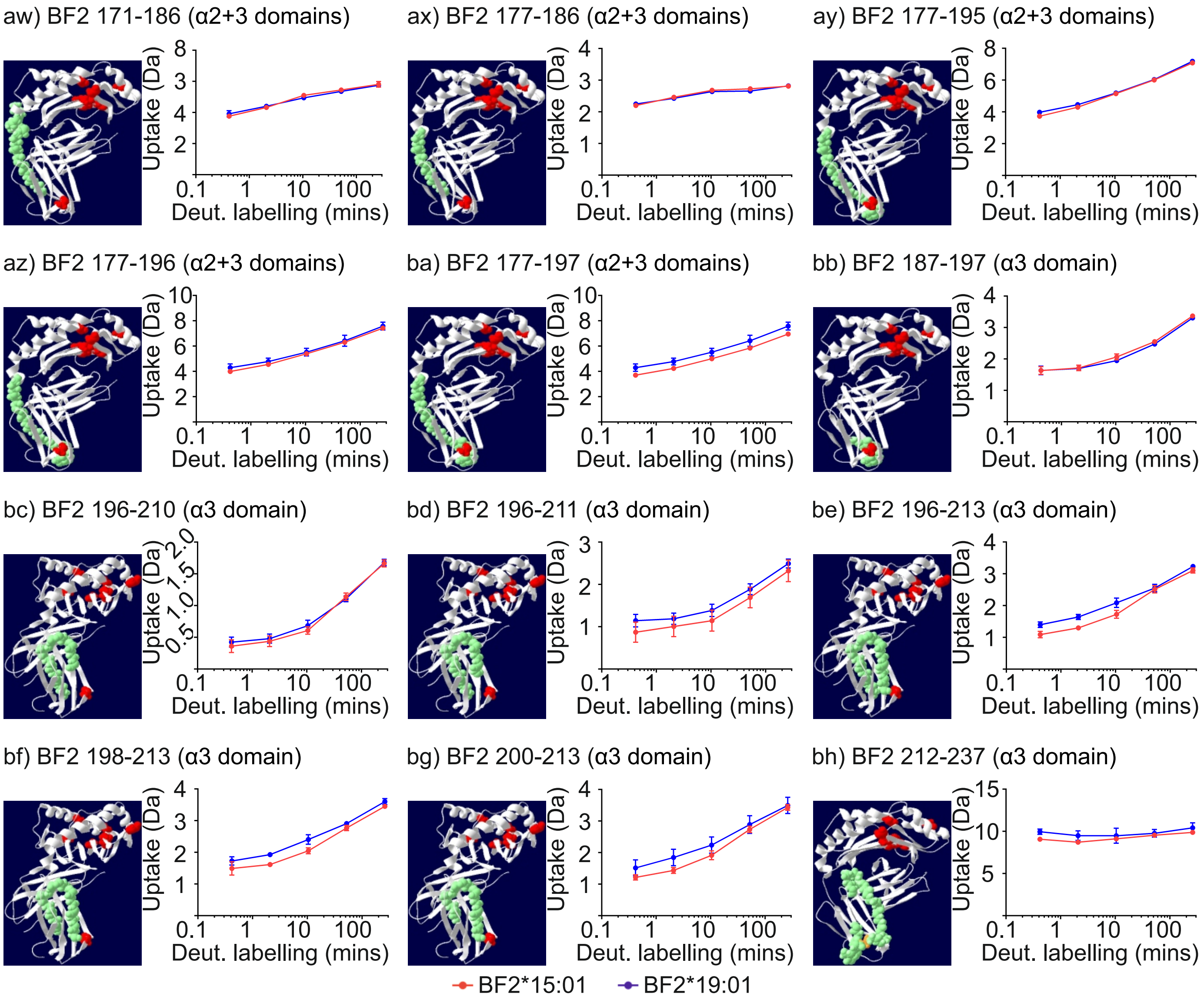

Supplementary figure 2

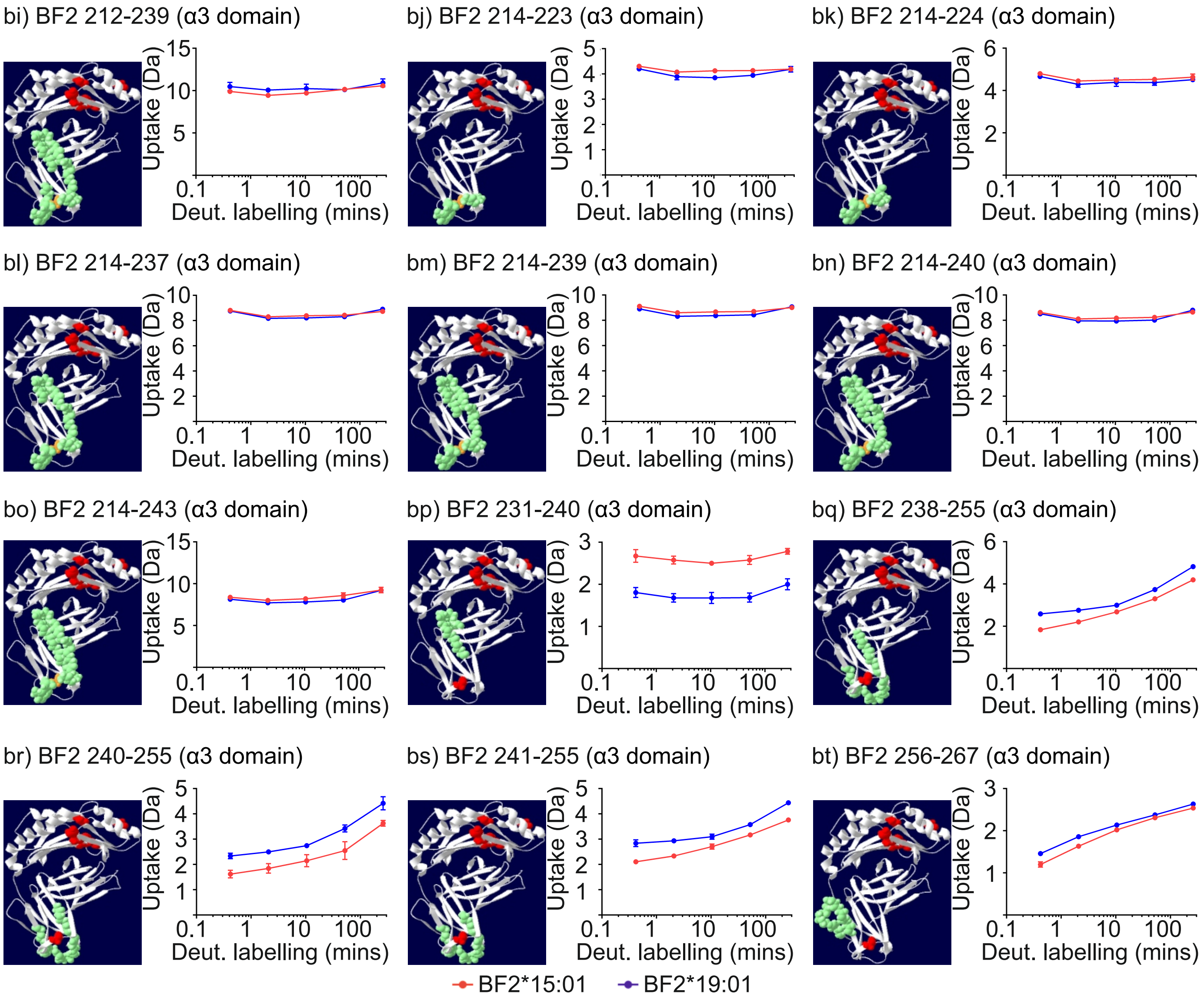

Supplementary figure 2 Comparison of deuterium uptake for all of the polypeptides derived from both BF2 allotypes in the conditional ligand-loaded state.

Comparison of the uptake of deuterium for each of the 72 polypeptides derived from both BF2 allotypes in the conditional ligand-loaded state (i.e. when hydrogen-deuterium exchange was initiated without prior UV exposure). The mean average deuterium uptake is plotted for each exposure time, with the standard deviation that was observed between replicates represented using vertical error bars. For each polypeptide the indicated sequence is shown in green based on a homology model of the BF2*15:01-KRLIGKRY complex. Polymorphic residues are shown in red, or in orange when the polymorphism is located within the polypeptide of interest.

Supplementary figure 3

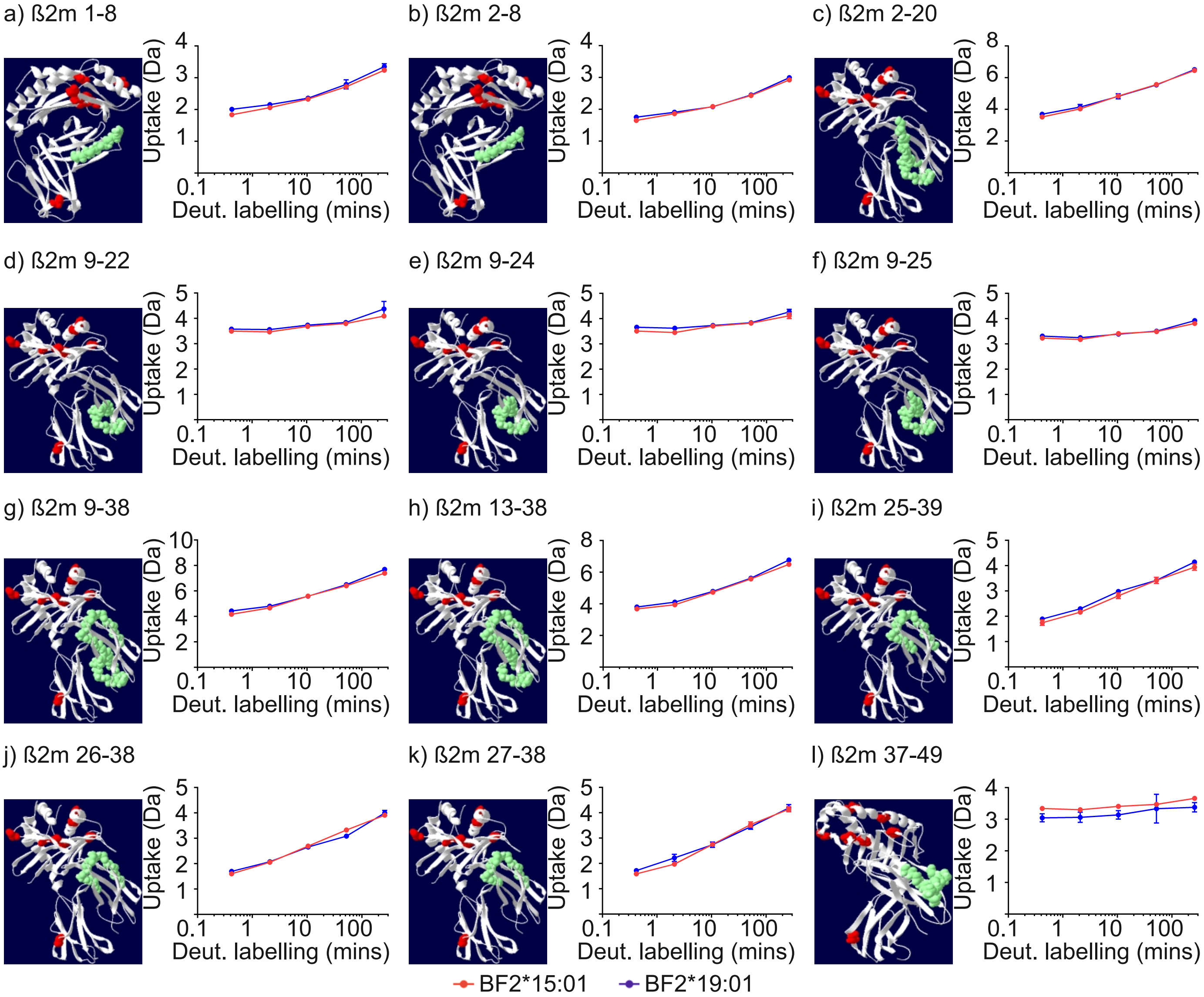

Supplementary figure 3

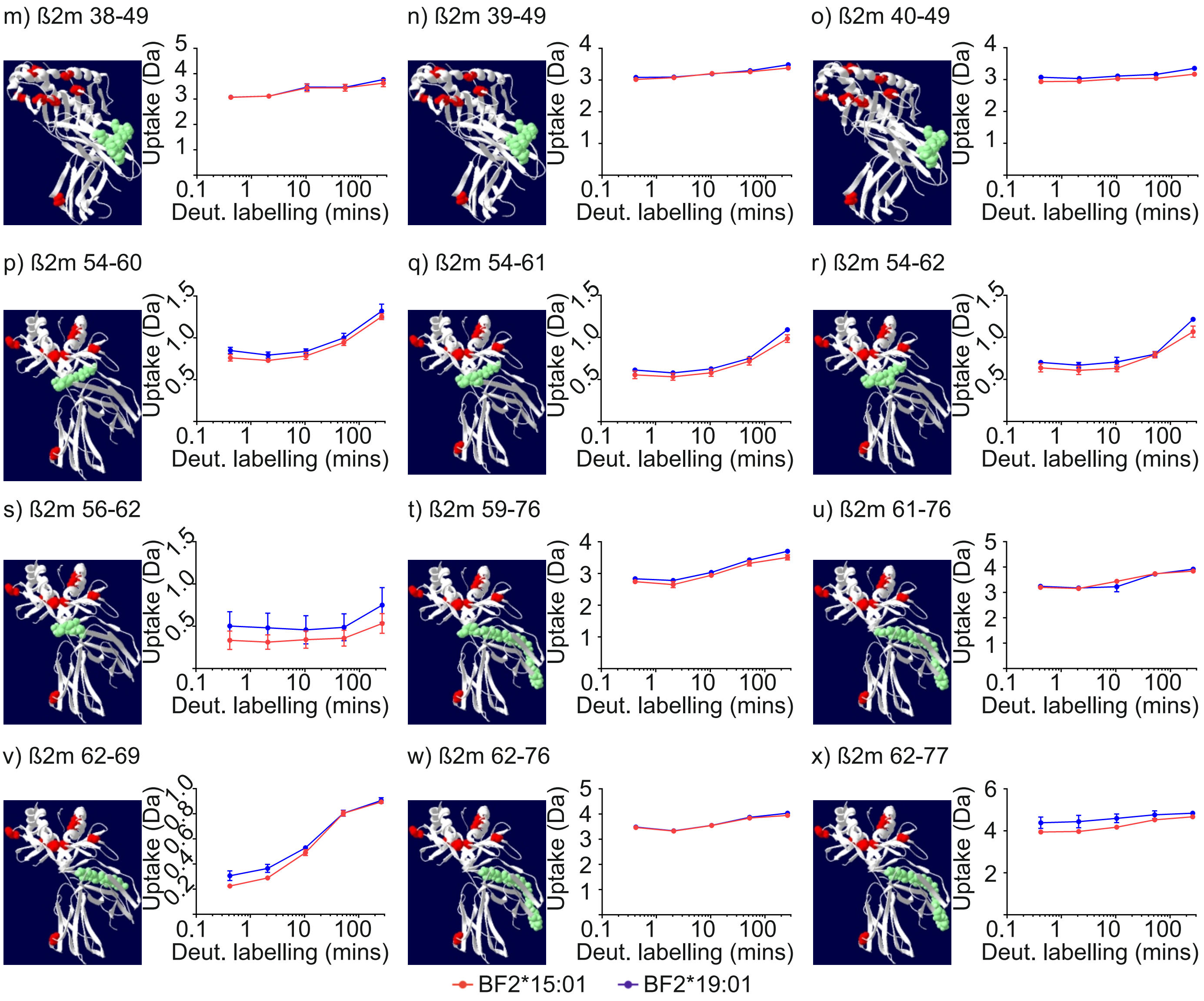

Supplementary figure 3

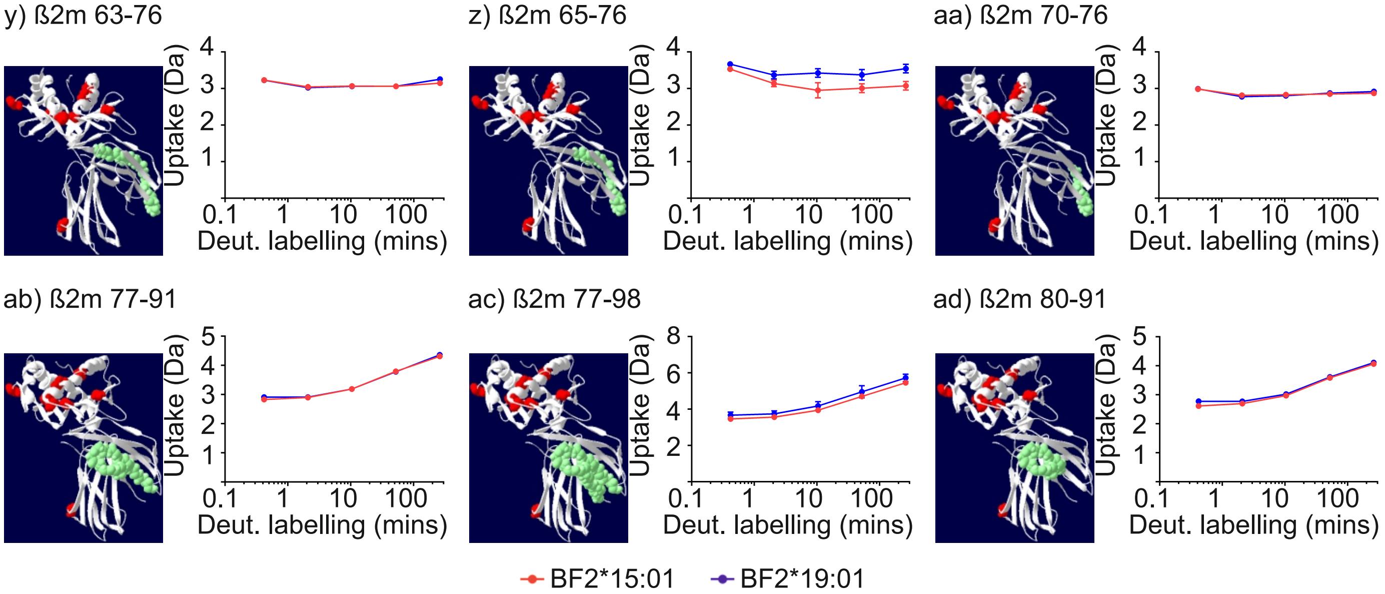

Supplementary figure 3 Comparison of deuterium uptake for all of the polypeptides derived from the β_2m molecules associated with the two BF2 allotypes in the conditional ligand-loaded state. Comparison of the uptake of deuterium for each of the 30 polypeptides derived from the β_2m molecules associated with both the BF2 allotypes in the conditional ligand-loaded state (i.e. when hydrogen-deuterium exchange was initiated without prior UV exposure), presented as in supplementary figure 2.

Supplementary figure 4

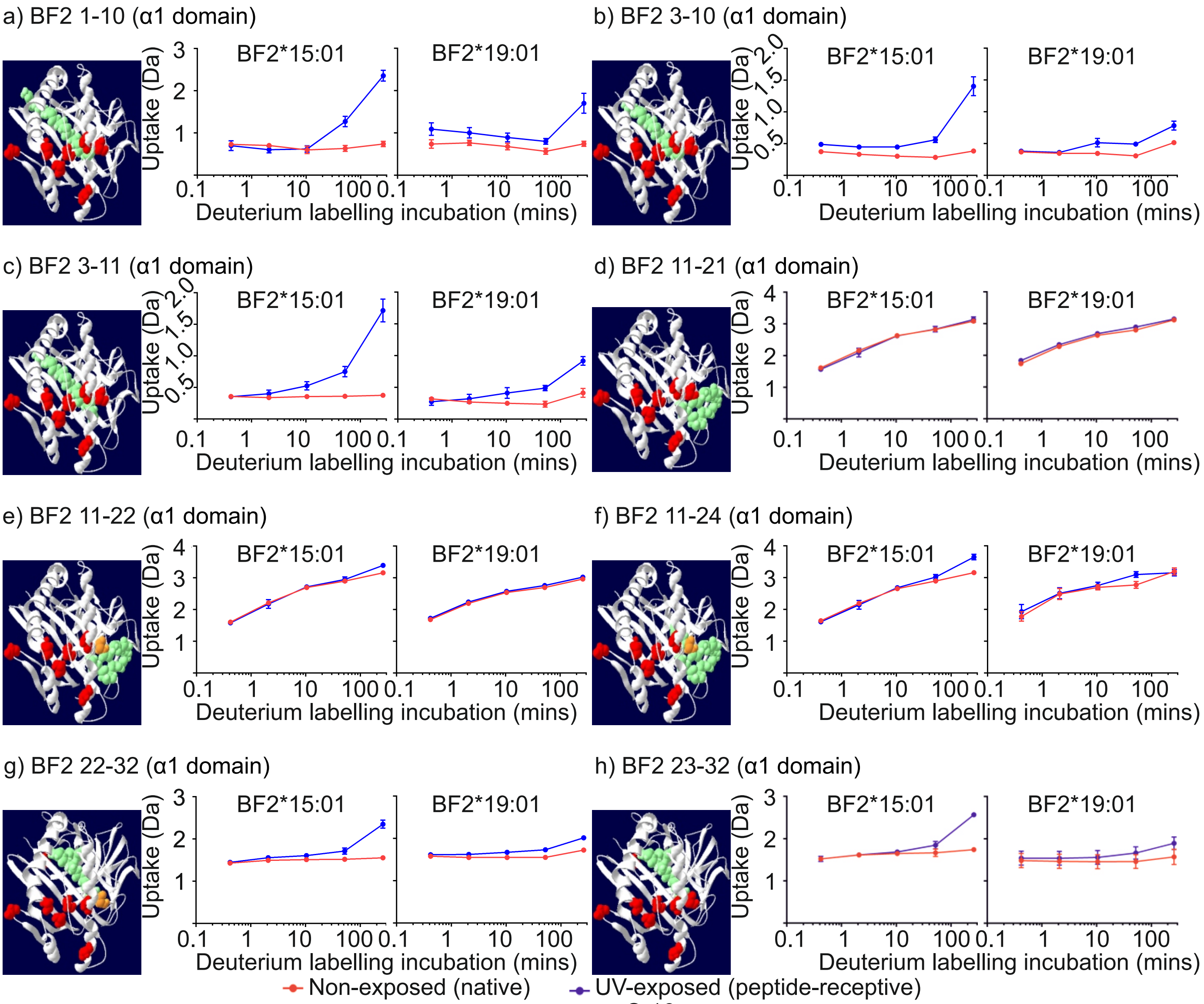

Supplementary figure 4

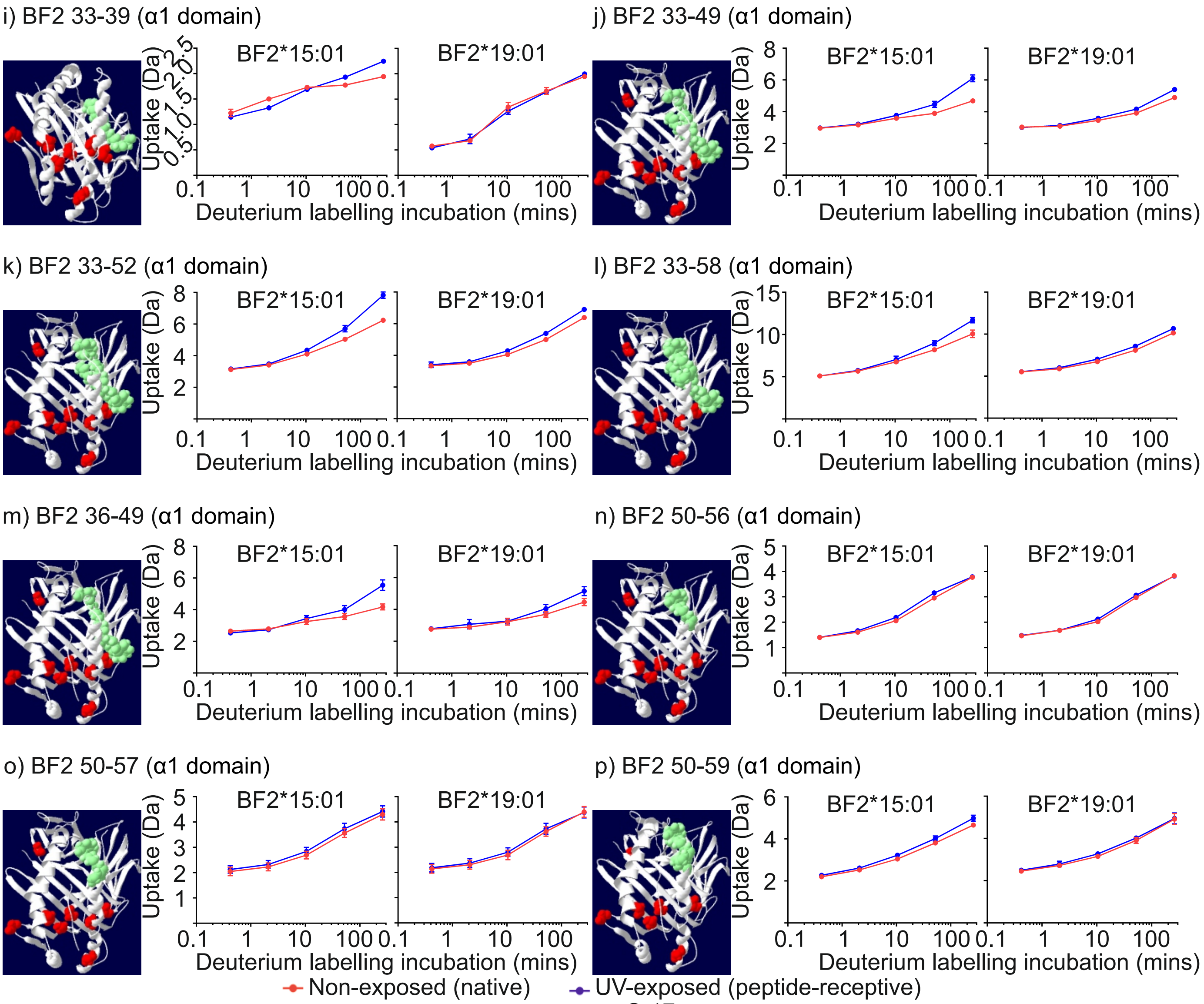

Supplementary figure 4

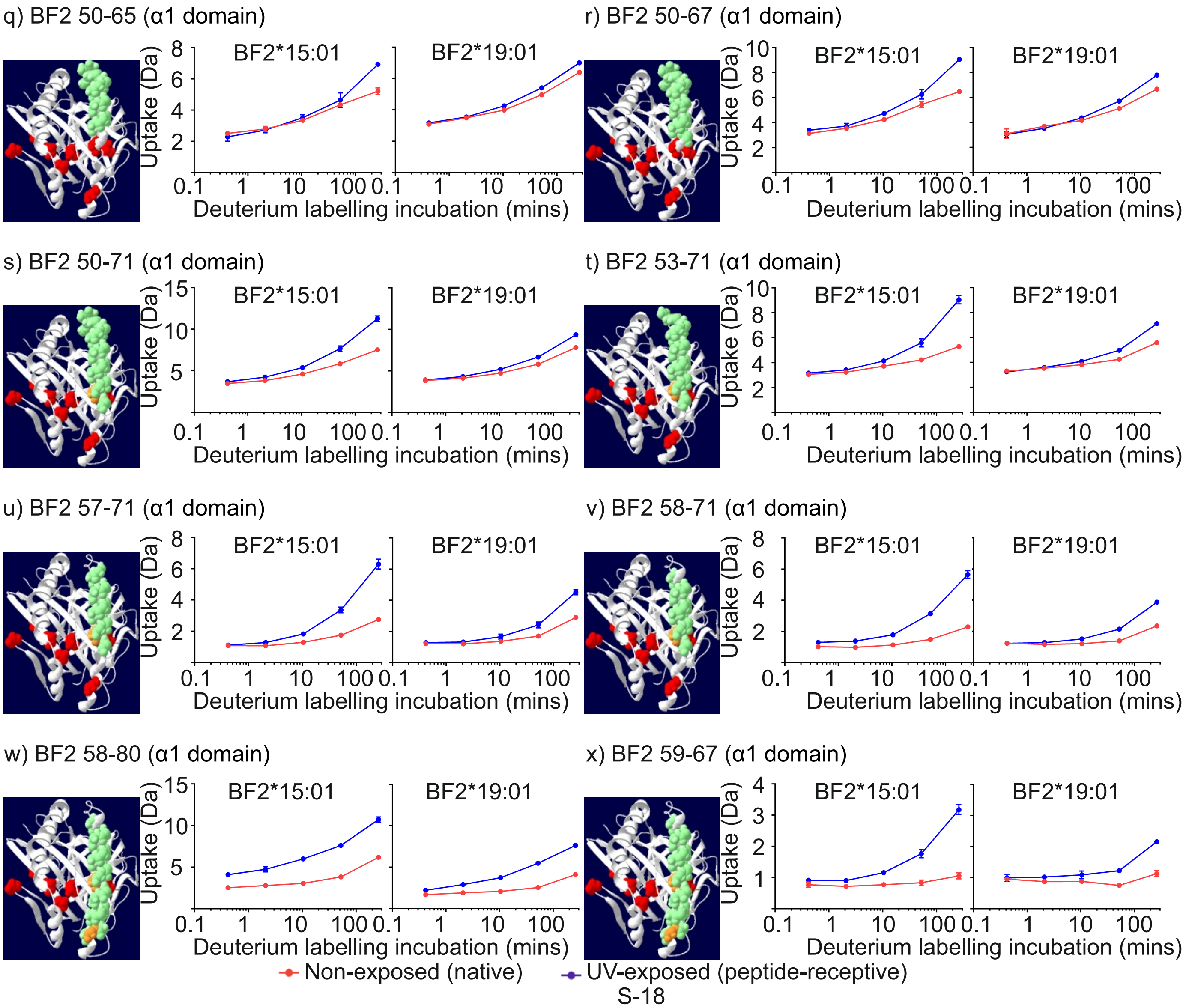

Supplementary figure 4

y) BF2 59-71 (α1 domain)

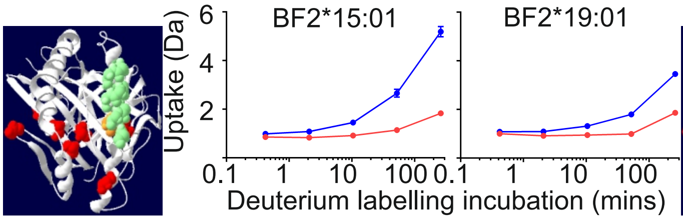

z) BF2 59-80 (α1 domain)

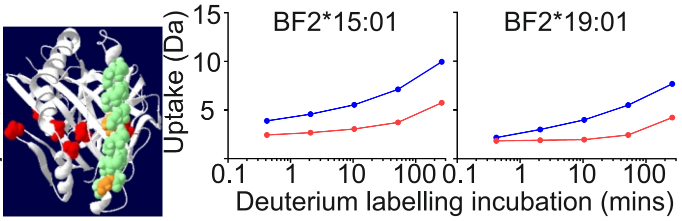

aa) BF2 60-71 (α1 domain)

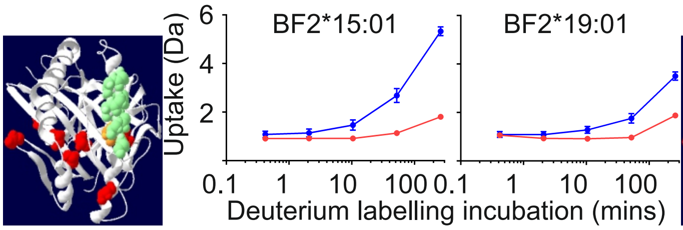

ab) BF2 68-80 (α1 domain)

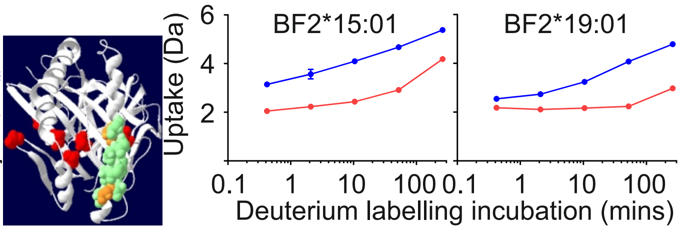

ac) BF2 81-90 (α1+2 domains)

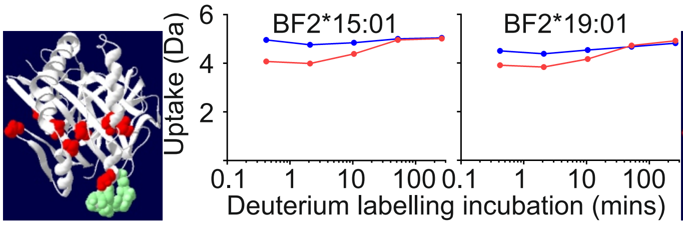

ad) BF2 81-94 (α1+2 domains)

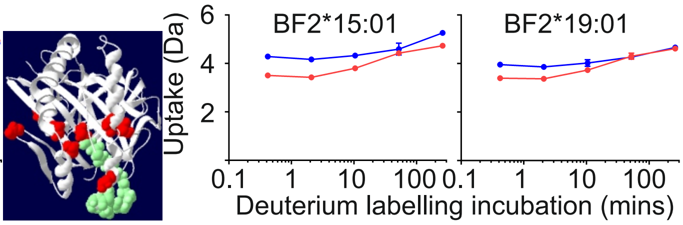

ae) BF2 81-95 (α1+2 domains)

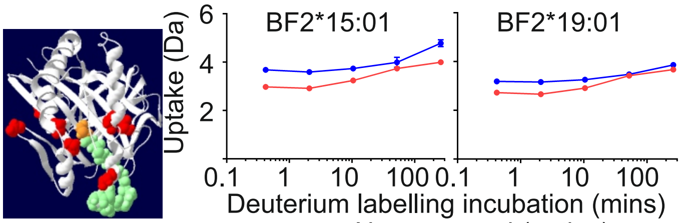

af) BF2 81-96 (α1+2 domains)

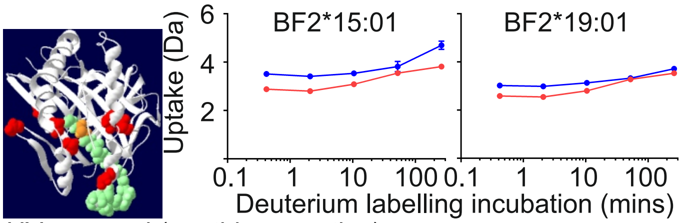

Non-exposed (native) UV-exposed (peptide-receptive)

Supplementary figure 4

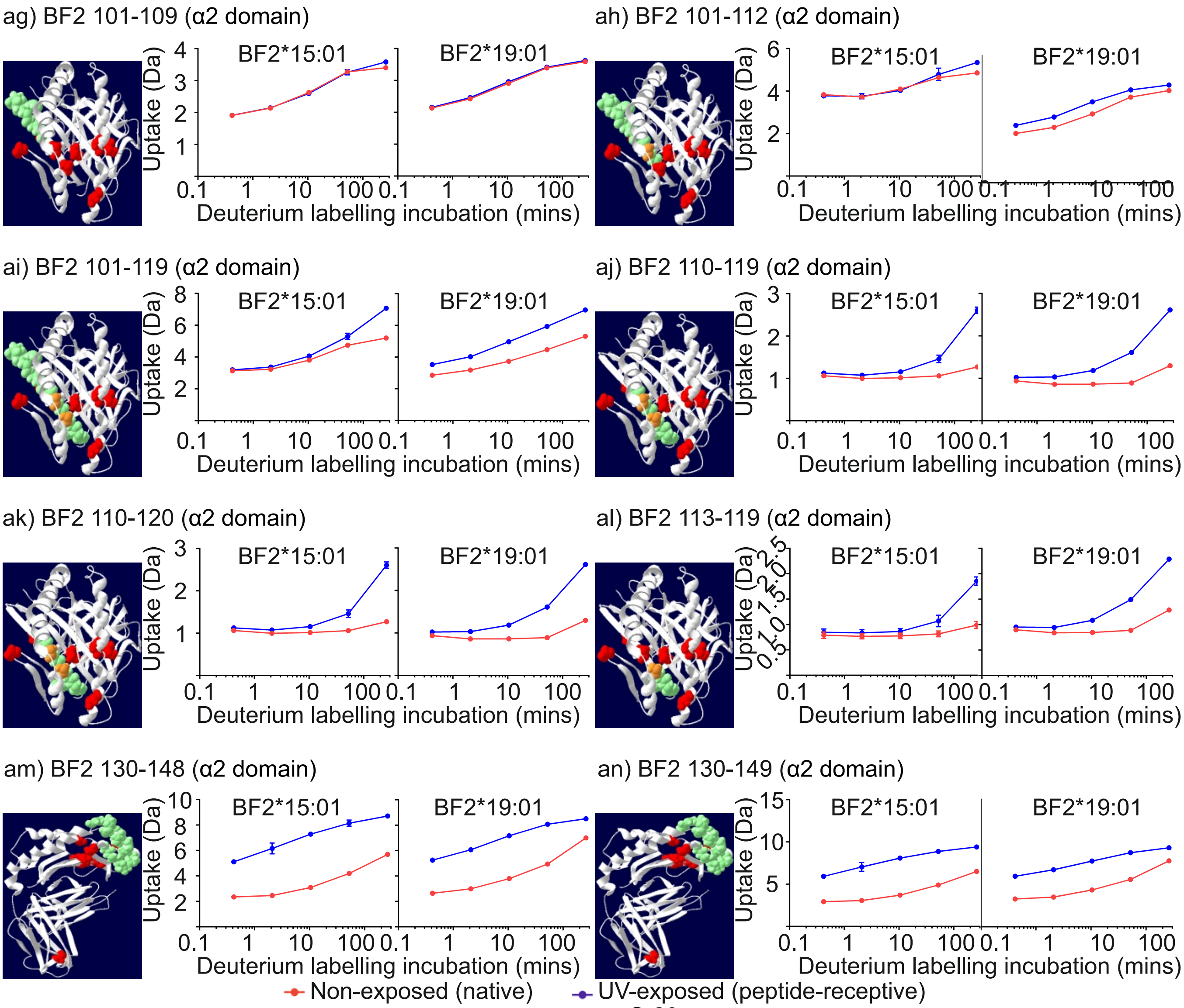

Supplementary figure 4

ao) BF2 131-148 (α2 domain)

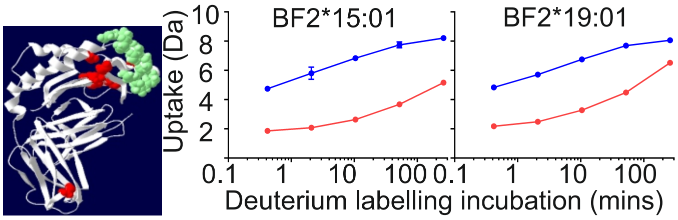

ap) BF2 131-149 (α2 domain)

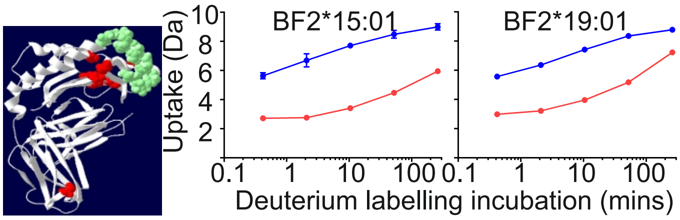

aq) BF2 131-155 (α2 domain)

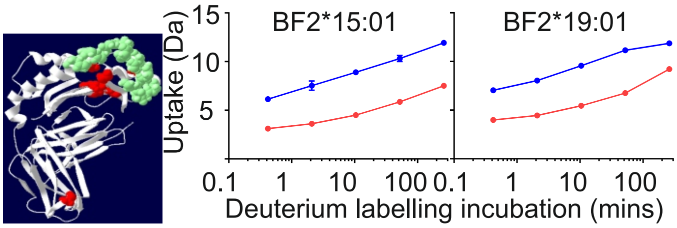

ar) BF2 133-148 (α2 domain)

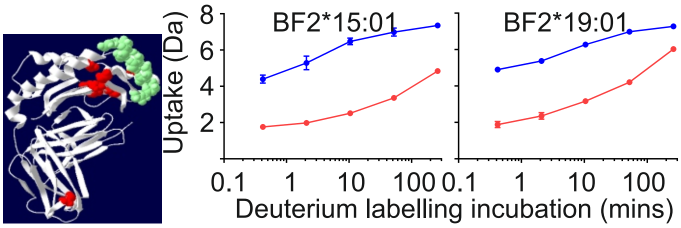

as) BF2 149-155 (α2 domain)

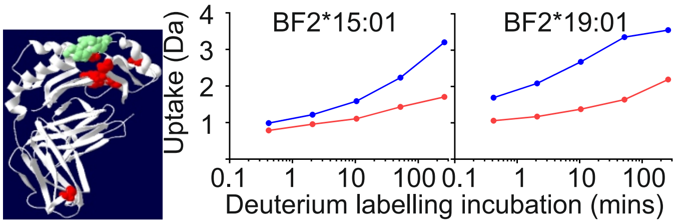

at) BF2 149-159 (α2 domain)

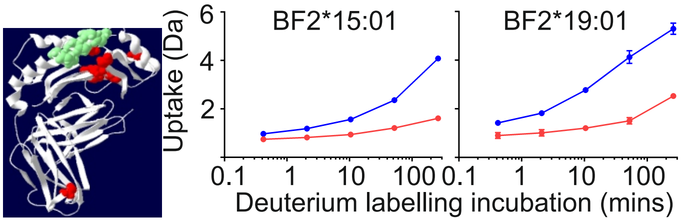

au) BF2 158-164 (α2 domain)

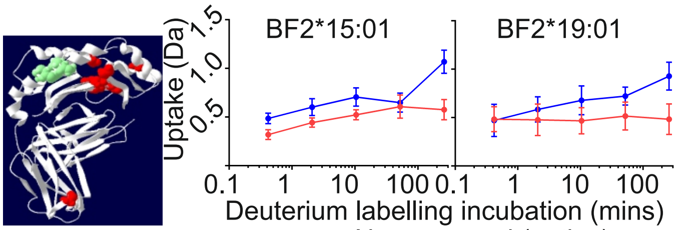

av) BF2 165-176 (α2 domain)

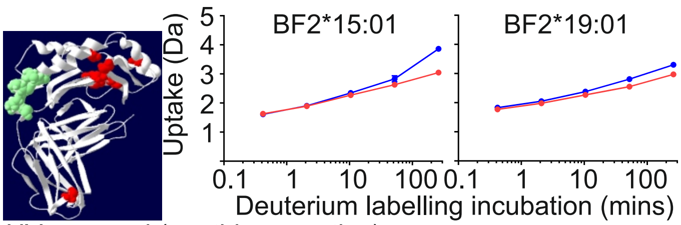

Non-exposed (native) UV-exposed (peptide-receptive)

Supplementary figure 4

aw) BF2 171-186 (α2+3 domains)

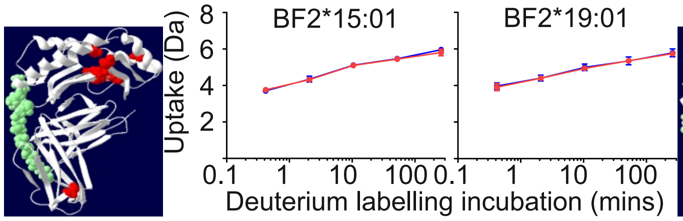

ax) BF2 177-186 (α2+3 domains)

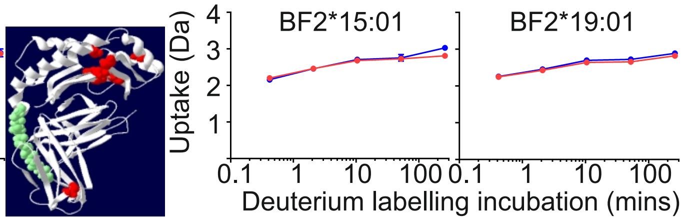

ay) BF2 177-195 (α2+3 domains)

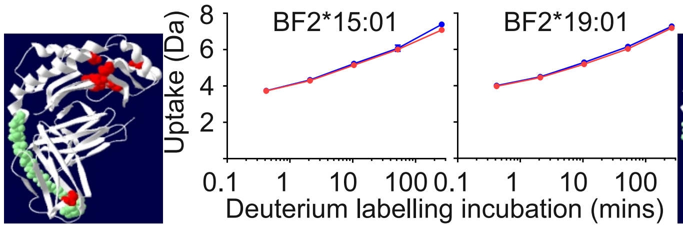

az) BF2 177-196 (α2+3 domains)

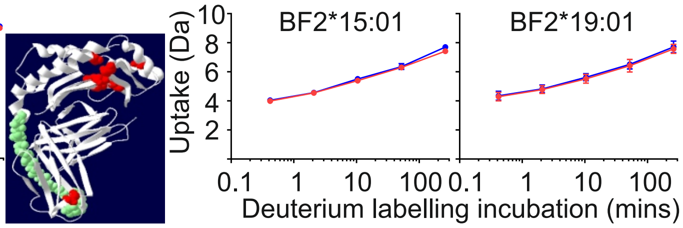

ba) BF2 177-197 (α2+3 domains)

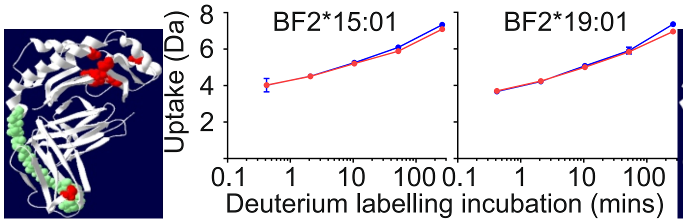

bb) BF2 187-197 (α3 domain)

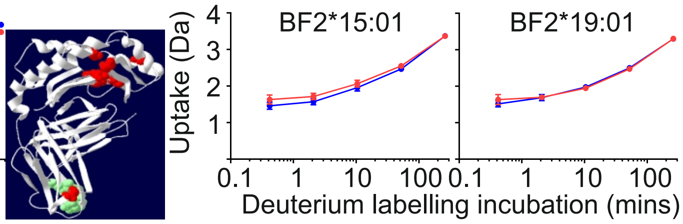

bc) BF2 196-210 (α3 domain)

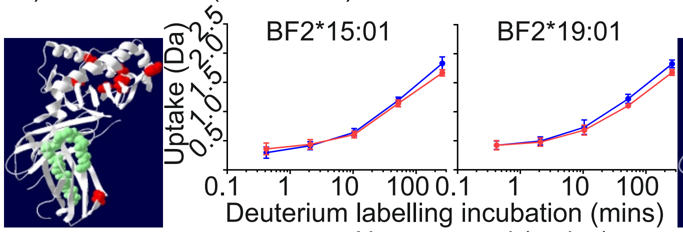

bd) BF2 196-211 (α3 domain)

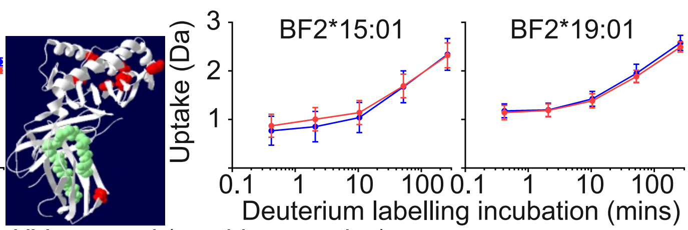

Non-exposed (native) UV-exposed (peptide-receptive)

Supplementary figure 4

be) BF2 196-213 (α3 domain)

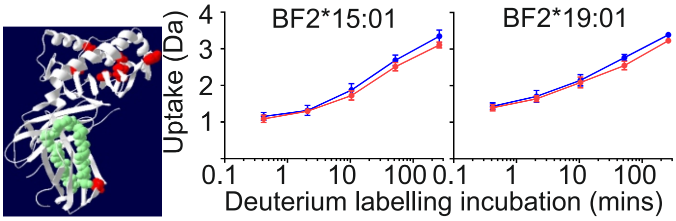

bf) BF2 198-213 (α3 domain)

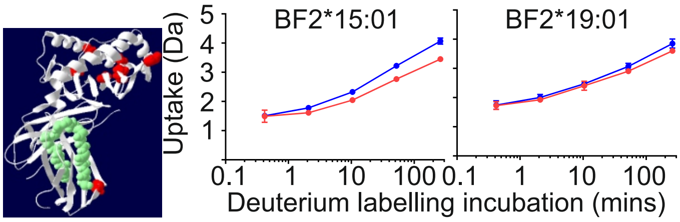

bg) BF2 200-213 (α3 domain)

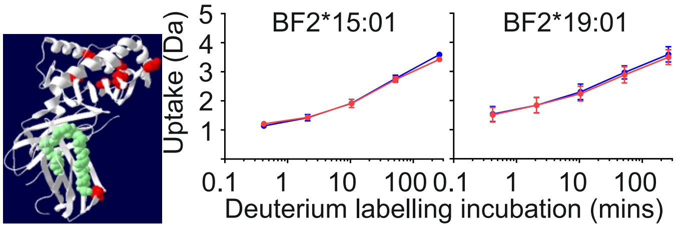

bh) BF2 212-237 (α3 domain)

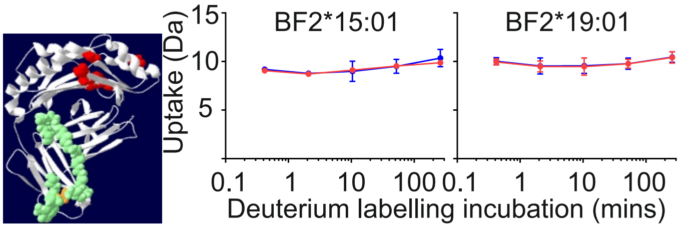

bi) BF2 212-239 (α3 domain)

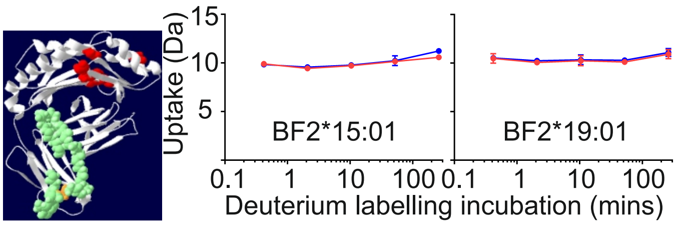

bj) BF2 214-223 (α3 domain)

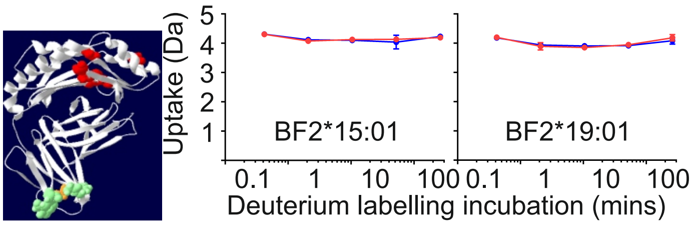

bk) BF2 214-224 (α3 domain)

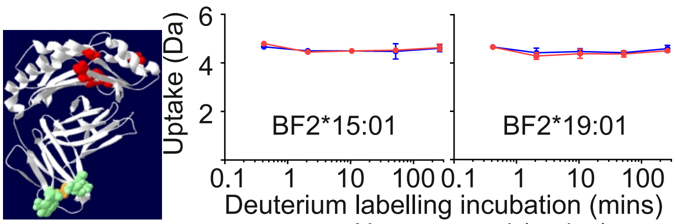

bl) BF2 214-237 (α3 domain)

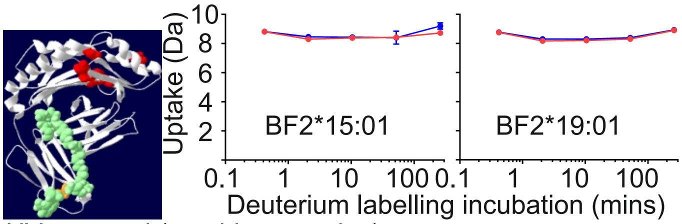

Non-exposed (native) UV-exposed (peptide-receptive)

Supplementary figure 4

bm) BF2 214-239 (α3 domain)

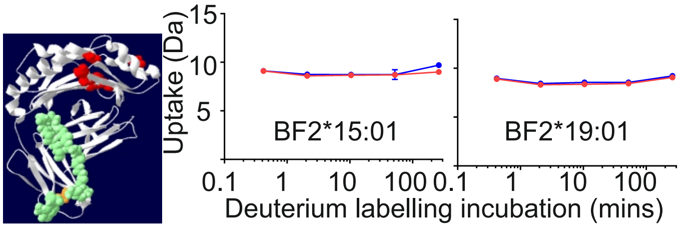

bn) BF2 214-240 (α3 domain)

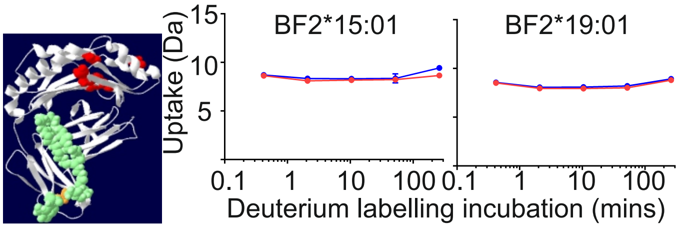

bo) BF2 214-243 (α3 domain)

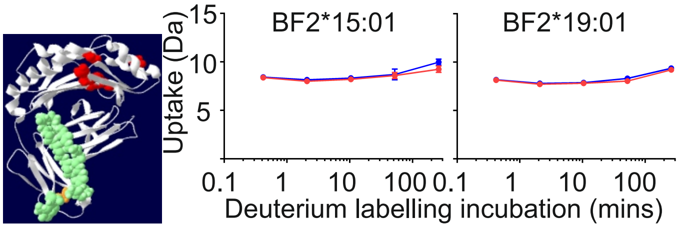

bp) BF2 231-240 (α3 domain)

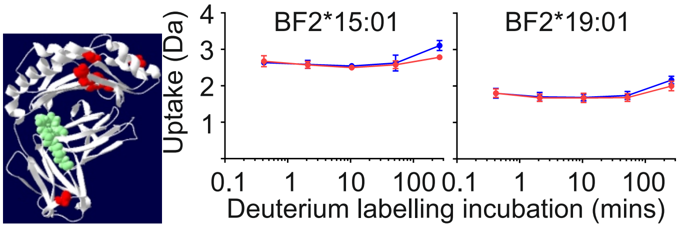

bq) BF2 238-255 (α3 domain)

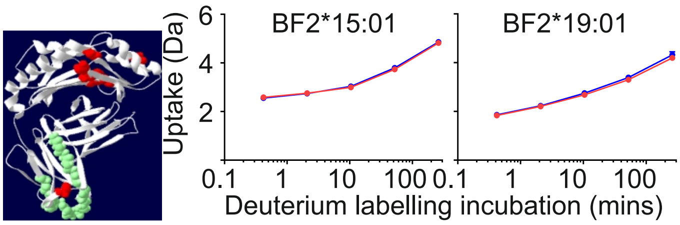

br) BF2 240-255 (α3 domain)

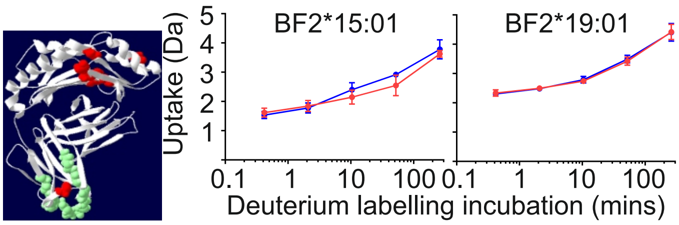

bs) BF2 241-255 (α3 domain)

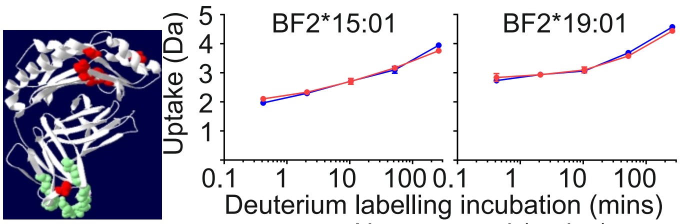

bt) BF2 256-267 (α3 domain)

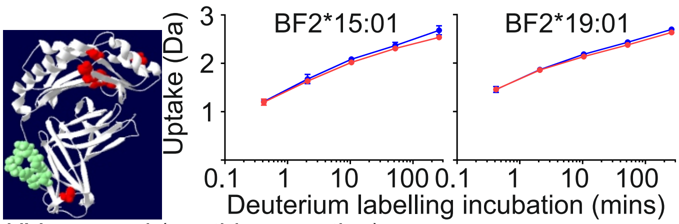

Non-exposed (native) UV-exposed (peptide-receptive)

Supplementary figure 4 Comparison of deuterium uptake for all of the polypeptides derived from both BF2 allotypes in the conditional ligand-loaded and peptide-receptive states.
Charts comparing the uptake of deuterium for each of the 72 polypeptides that were obtained from both of the BF2 allotypes in either the conditional ligand-loaded or peptide-receptive states. The mean average deuterium uptake is plotted for each exposure time and protein state, with the standard deviation that was observed between replicates represented using vertical error bars.

Supplementary figure 5

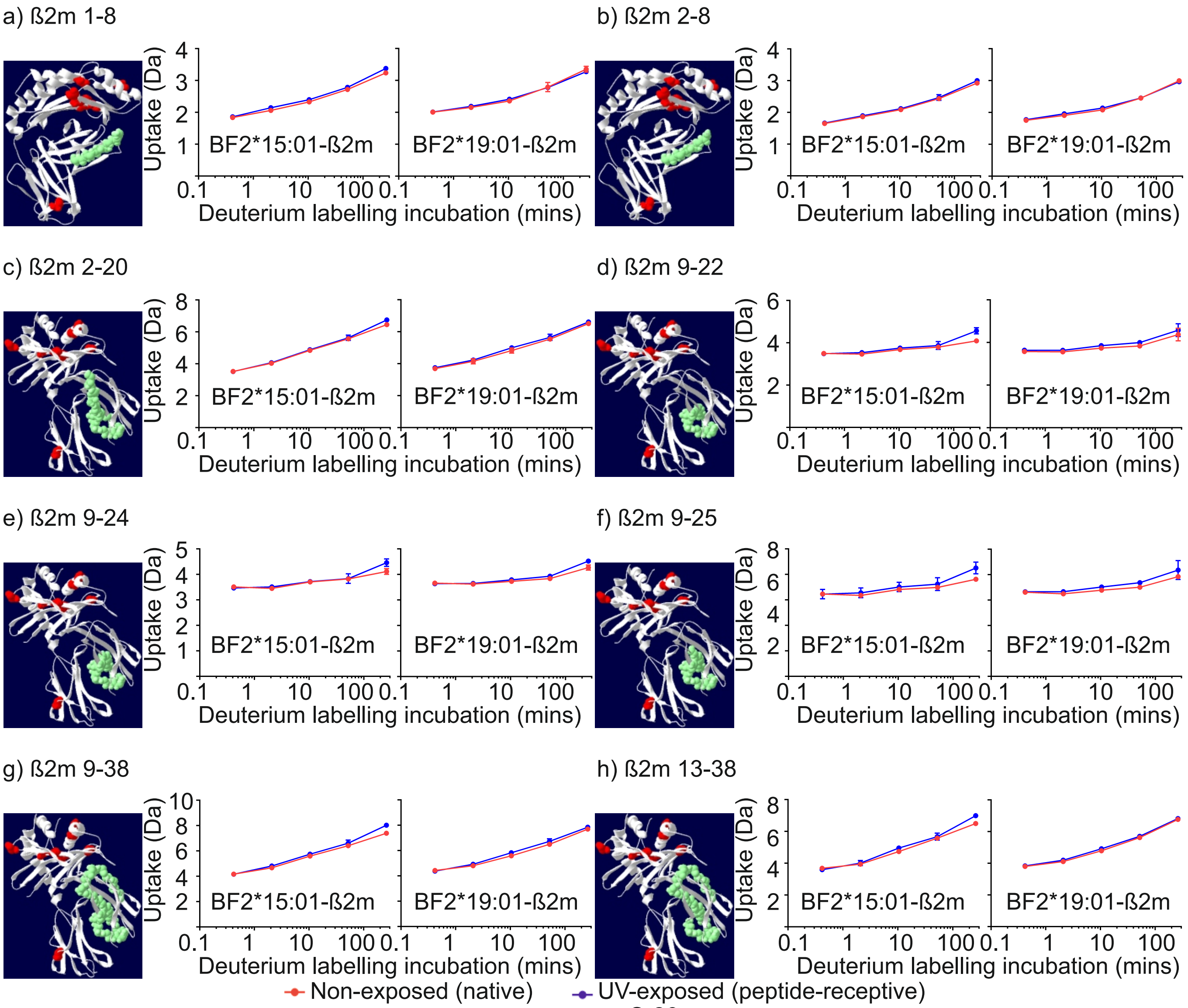

Supplementary figure 5

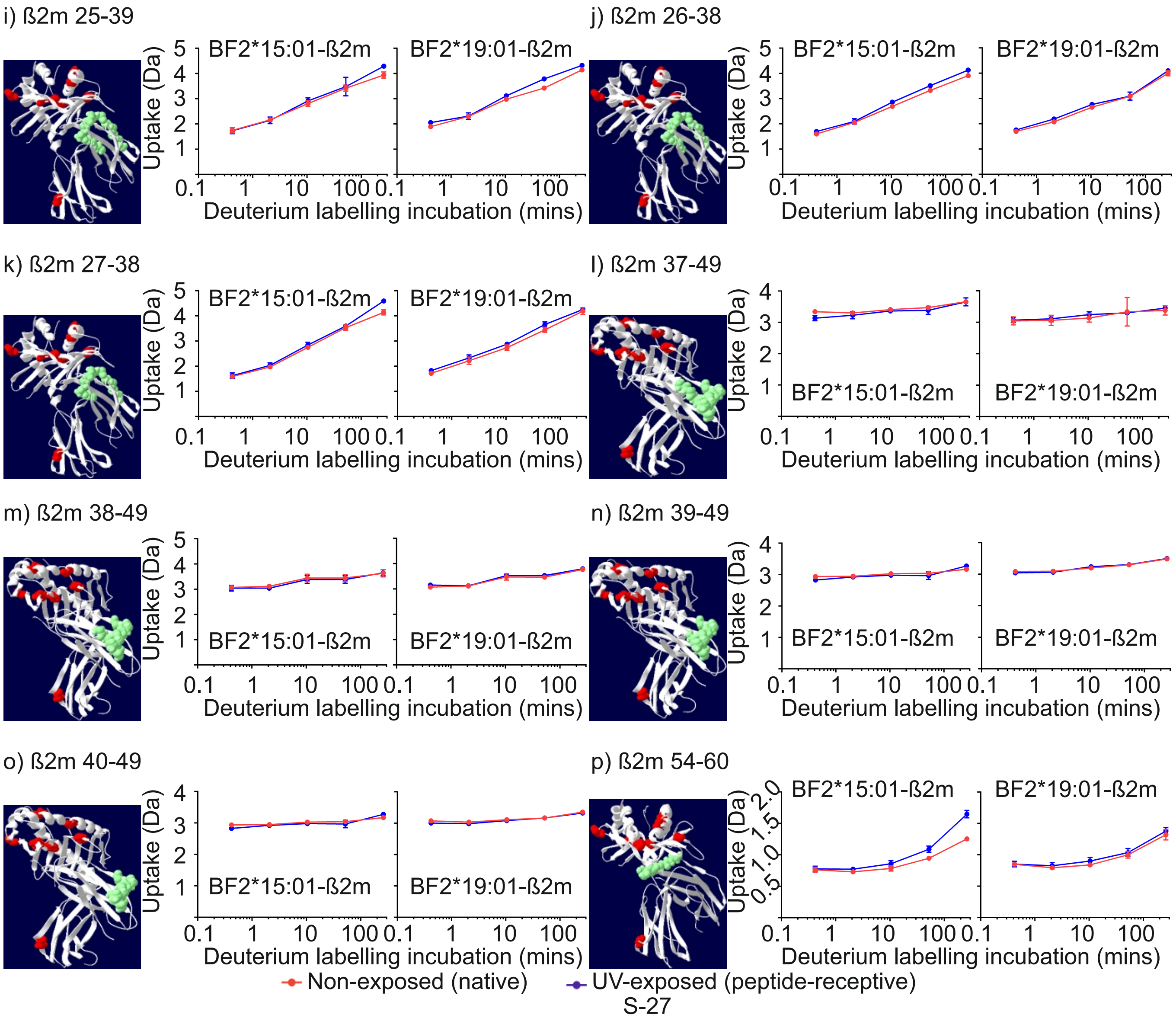

Supplementary figure 5

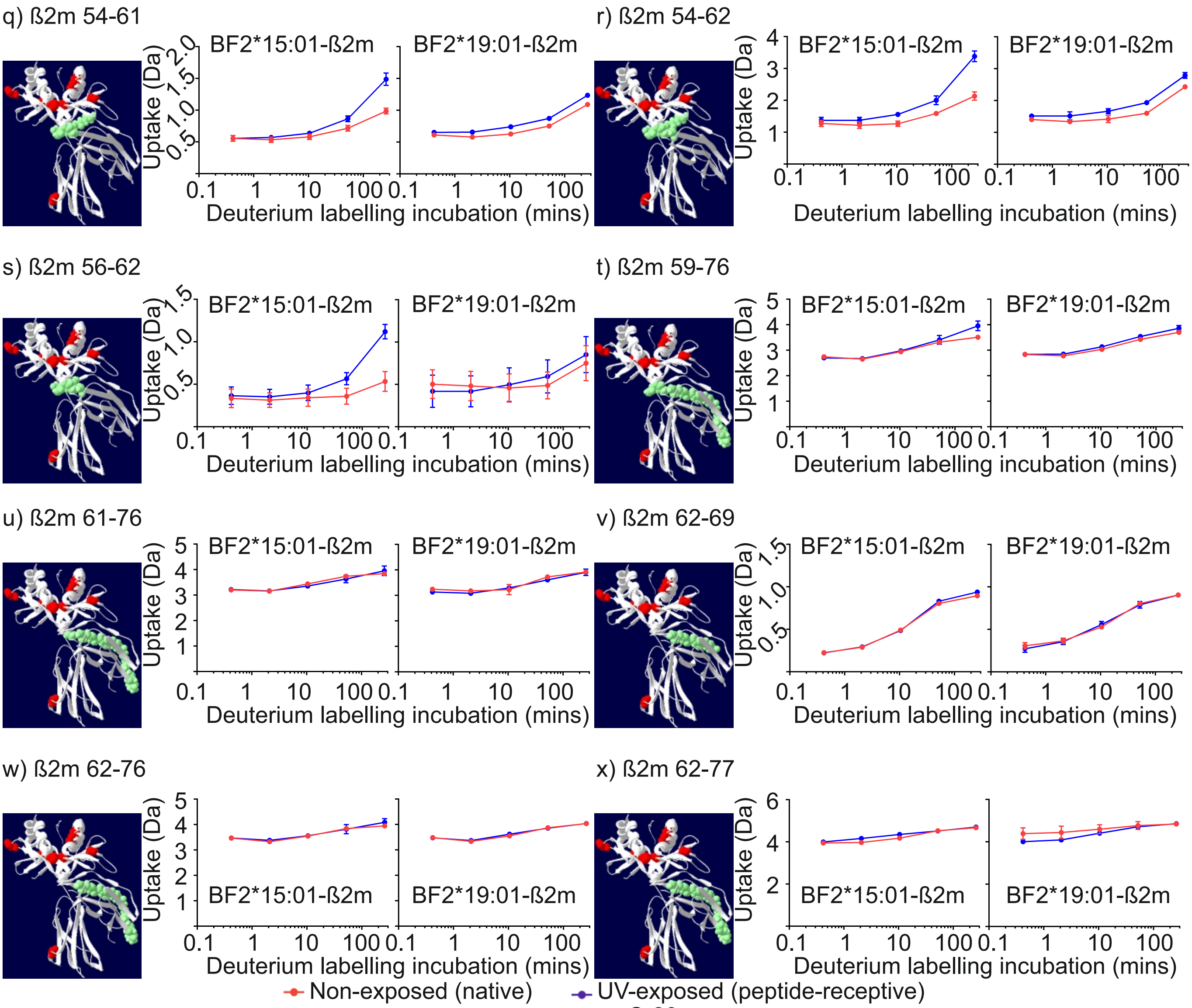

Supplementary figure 5

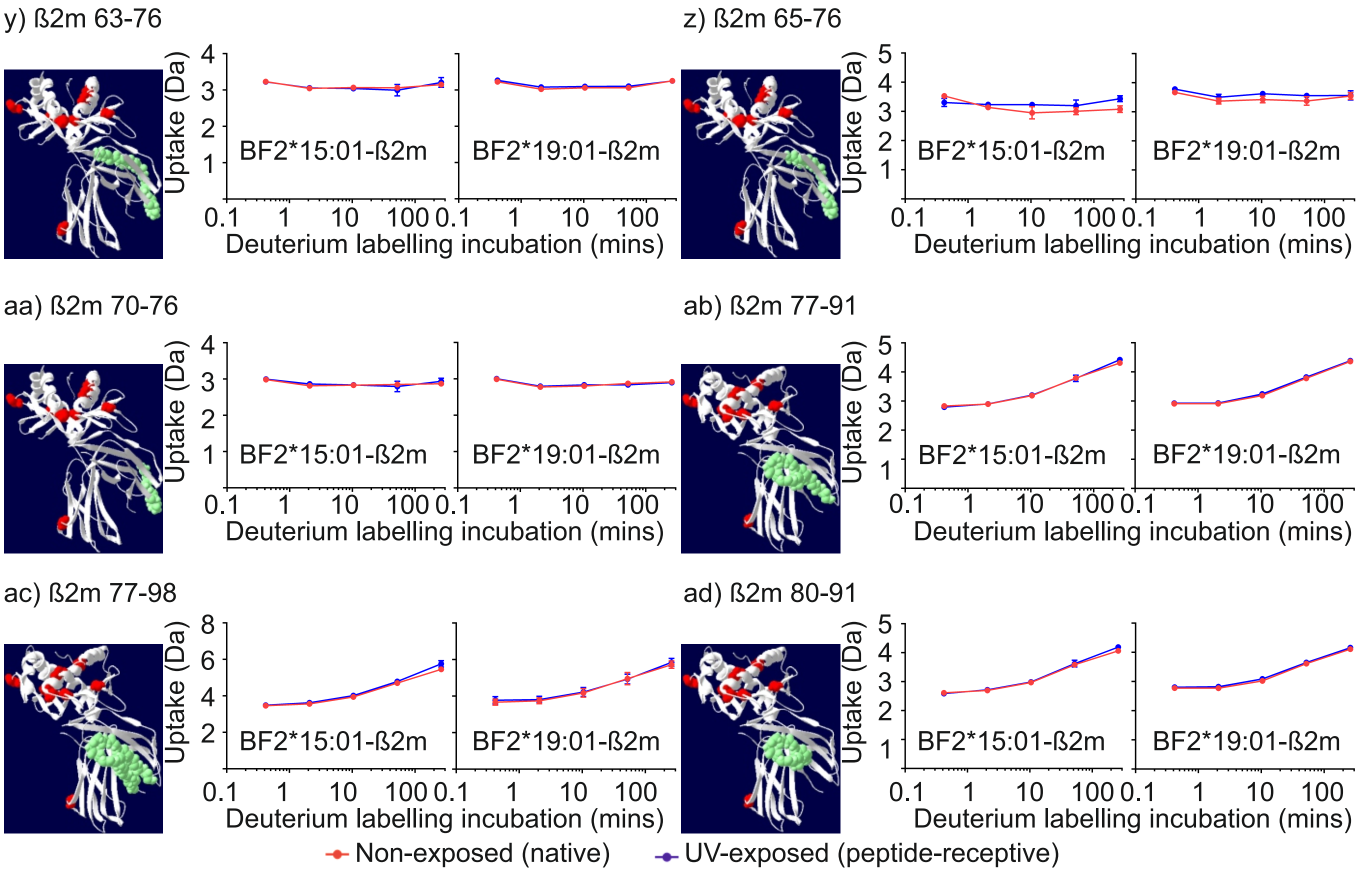

Supplementary figure 5 Comparison of deuterium uptake for all of the polypeptides derived from the β2m molecules associated with the two BF2 allotypes in the conditional ligand-loaded and peptide-receptive states.

Charts comparing the uptake of deuterium for each of the 30 polypeptides that were obtained from the β2m molecules associated with both of the BF2 allotypes in either the conditional ligand-loaded or peptide-receptive states, presented as in supplementary figure 4.

Supplementary figure 6

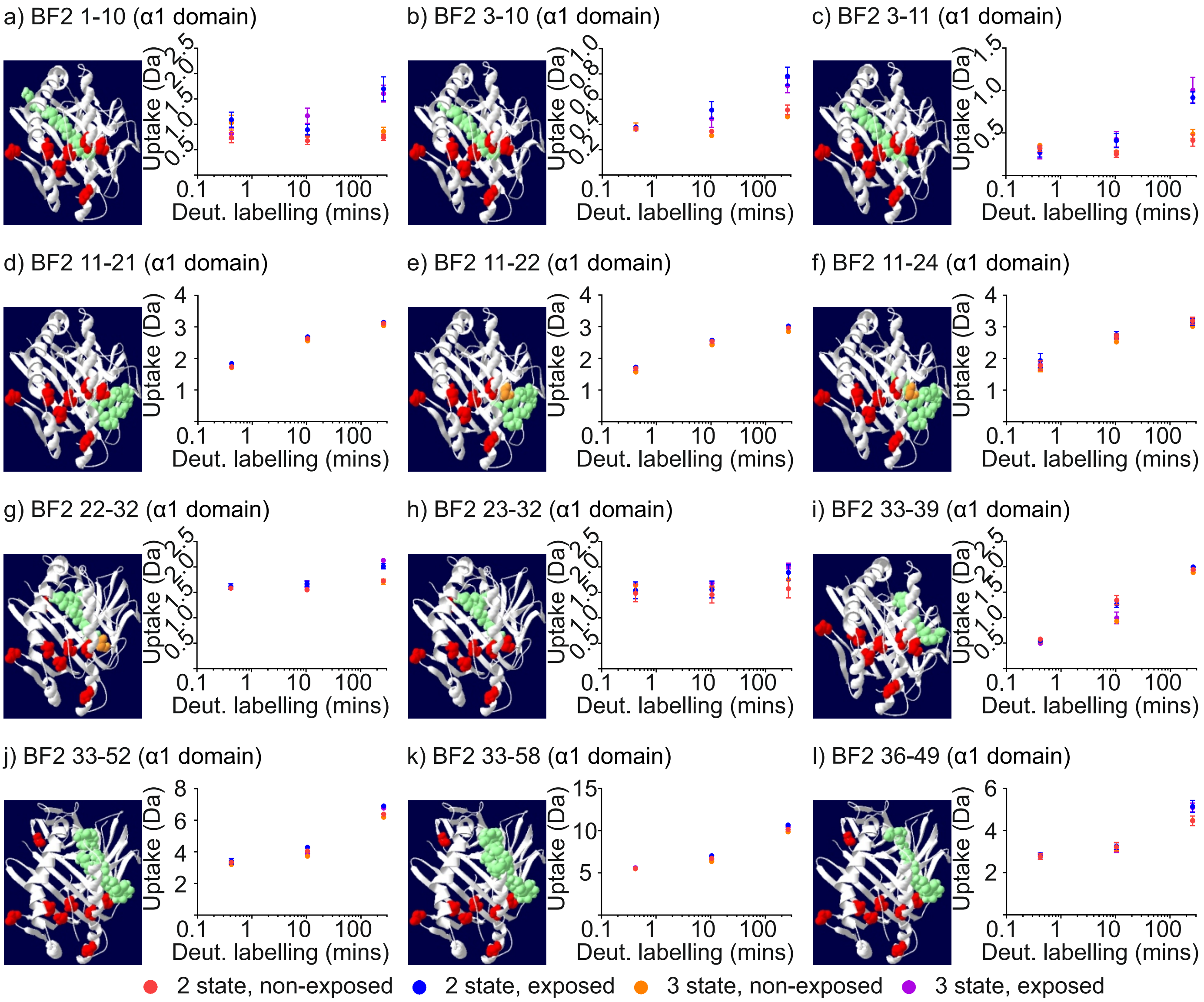

Supplementary figure 6

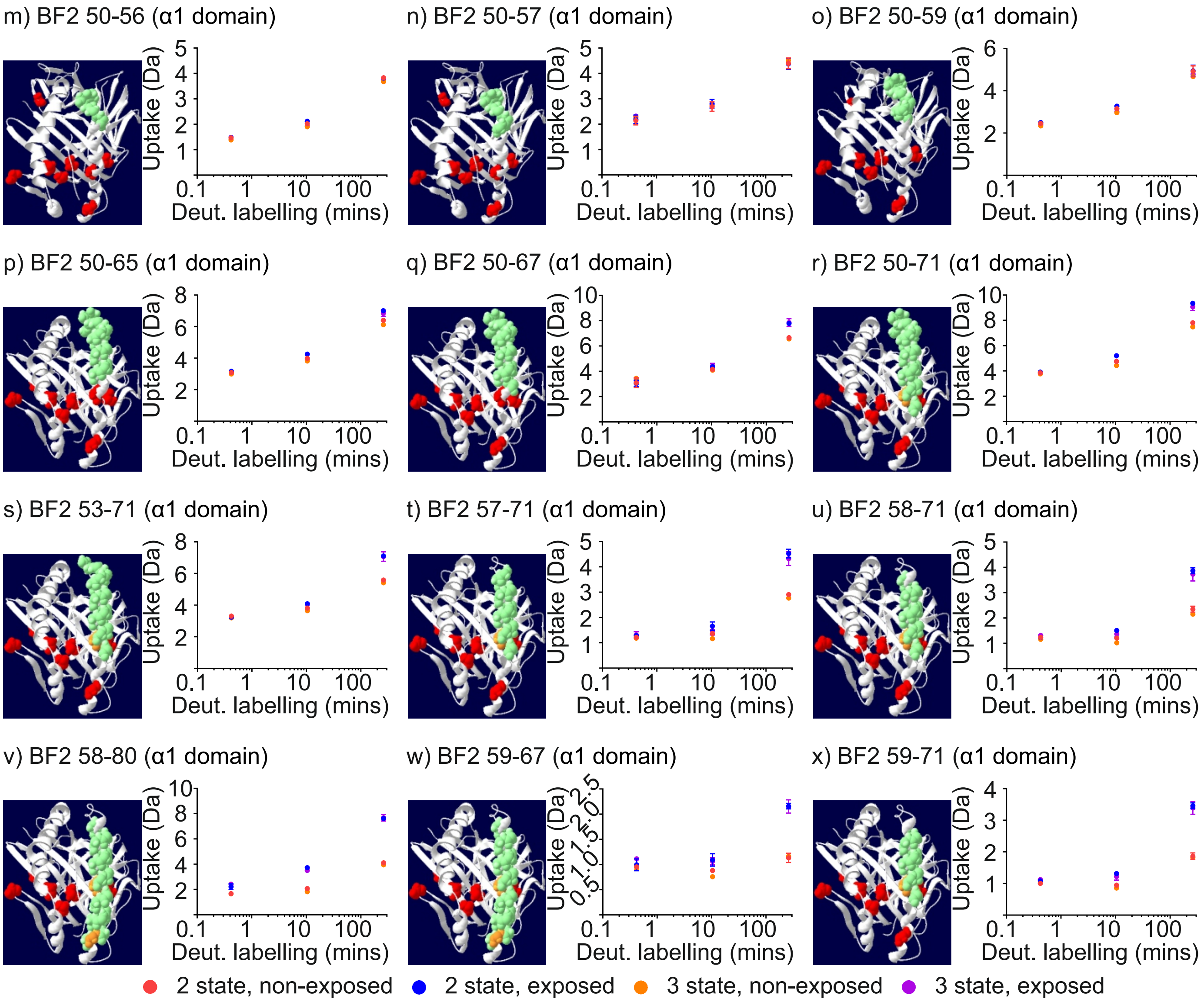

Supplementary figure 6

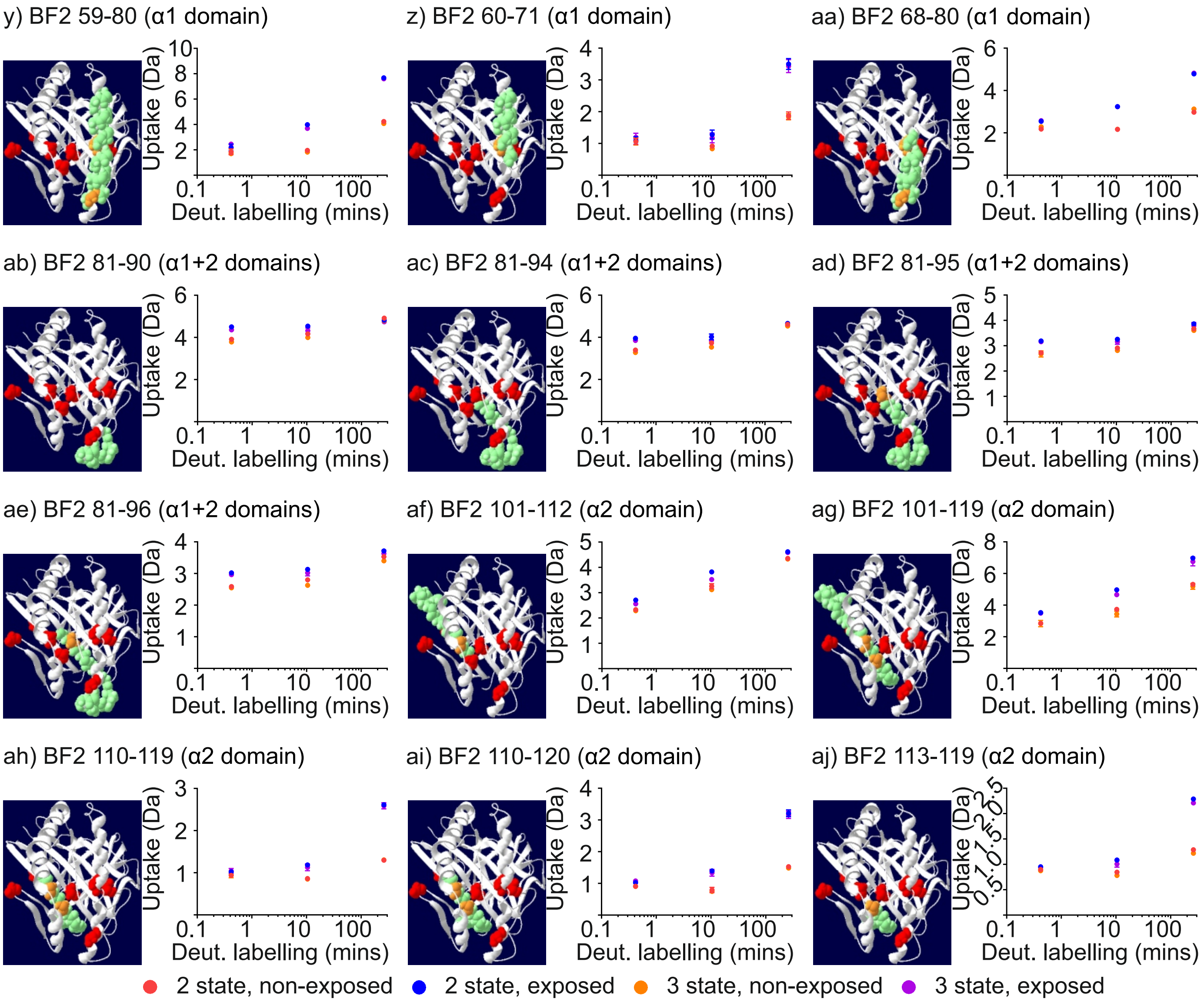

Supplementary figure 6

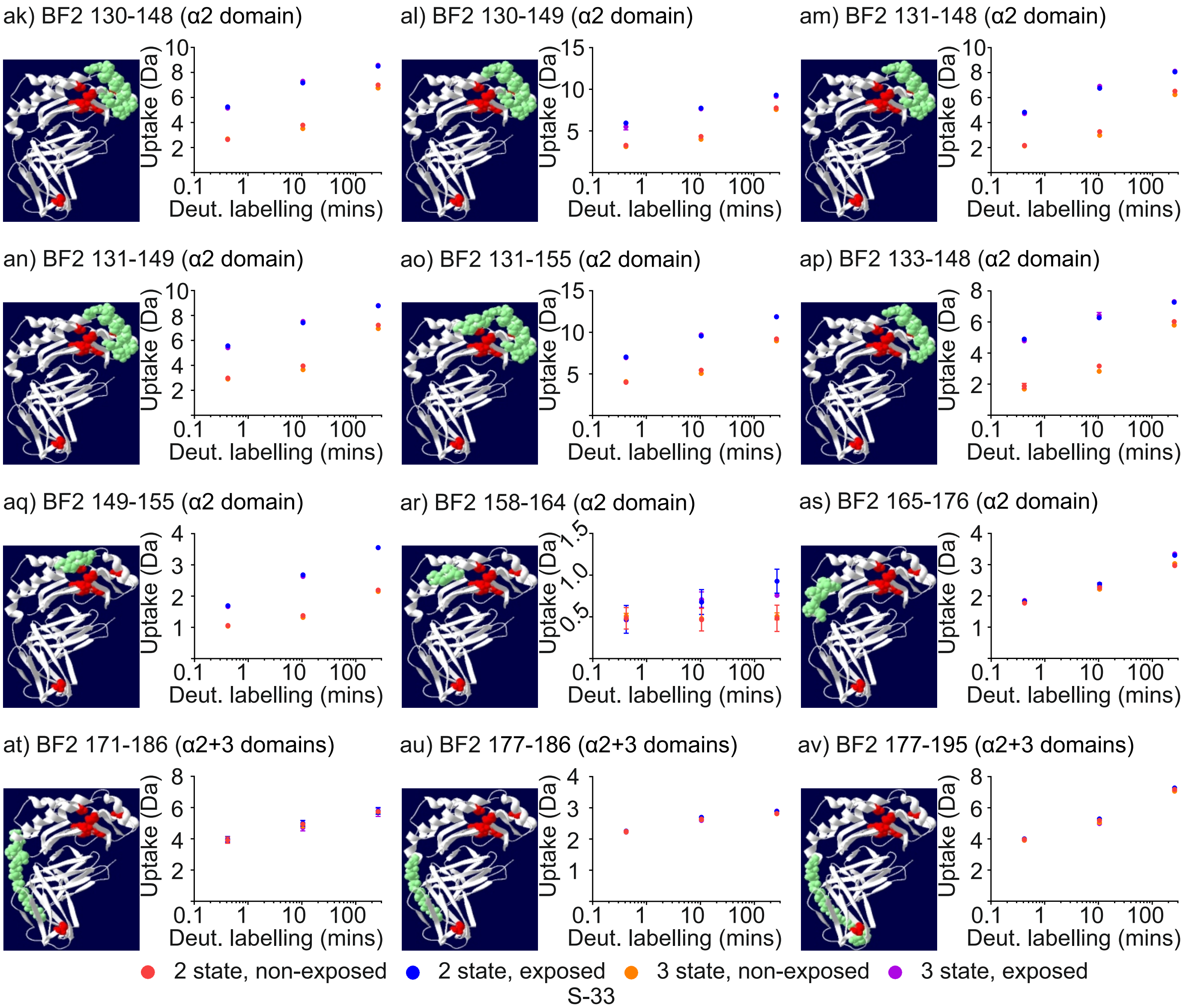

Supplementary figure 6

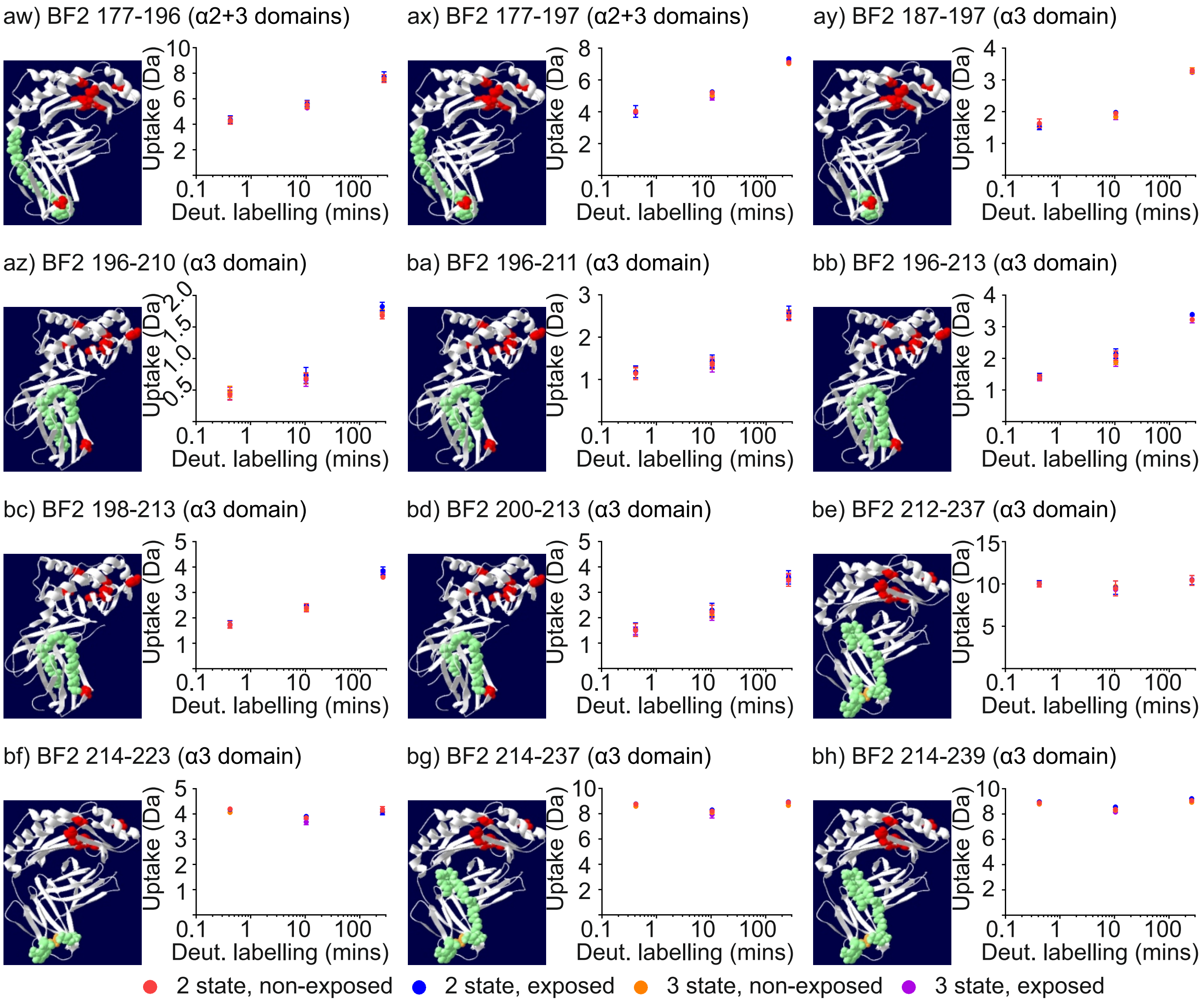

Supplementary figure 6

bi) BF2 214-240 (α3 domain)

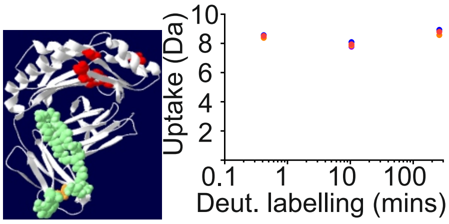

bj) BF2 214-243 (α3 domain)

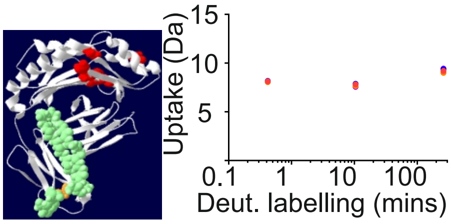

bk) BF2 231-240 (α3 domain)

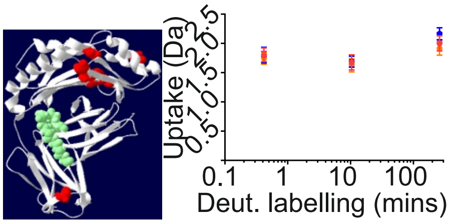

bl) BF2 238-255 (α3 domain)

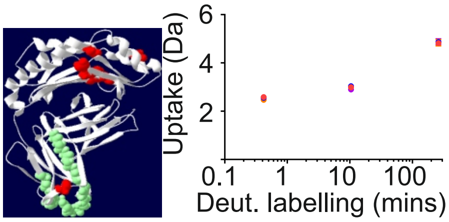

bm) BF2 240-255 (α3 domain)

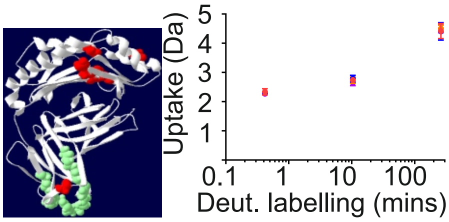

bn) BF2 241-255 (α3 domain)

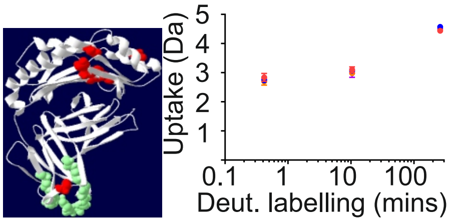

bo) BF2 256-267 (α3 domain)

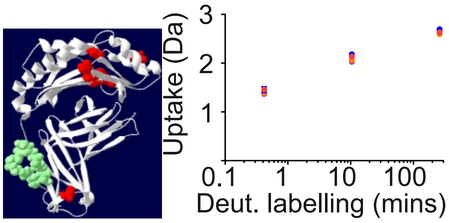

bp) ß2m 1-8

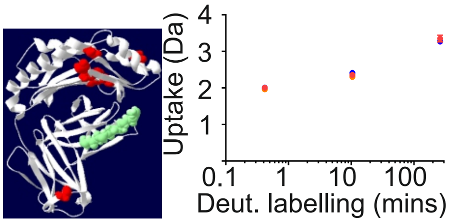

bq) ß2m 2-8

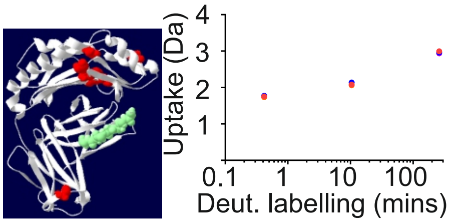

br) ß2m 9-22

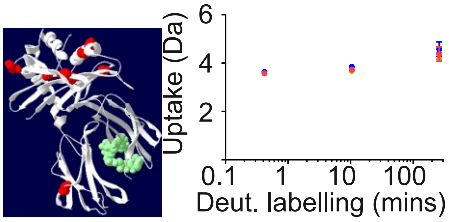

bs) ß2m 9-24

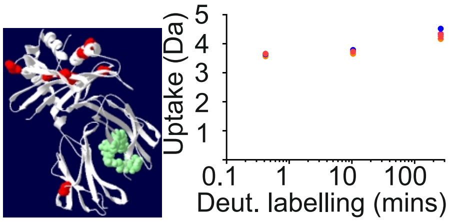

bt) ß2m 9-25

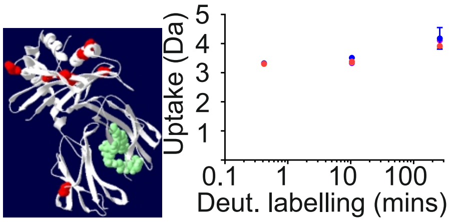

● 2 state, non-exposed ● 2 state, exposed ● 3 state, non-exposed ● 3 state, exposed

Supplementary figure 6

bu) ß2m 9-38

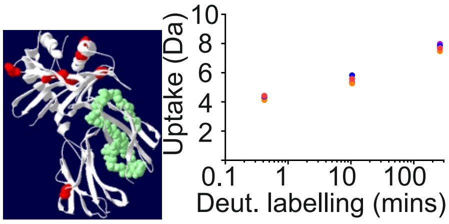

bv) ß2m 13-38

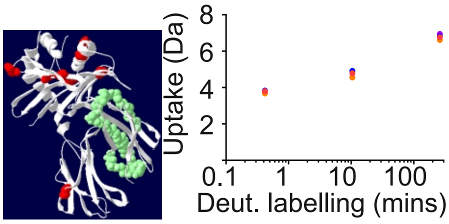

bw) ß2m 25-39

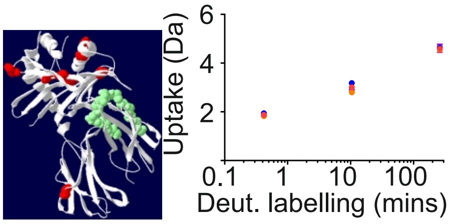

bx) ß2m 26-38

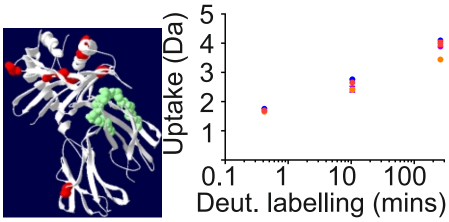

by) ß2m 27-38

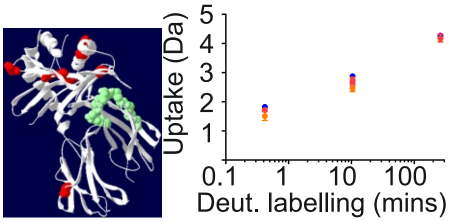

bz) ß2m 37-49

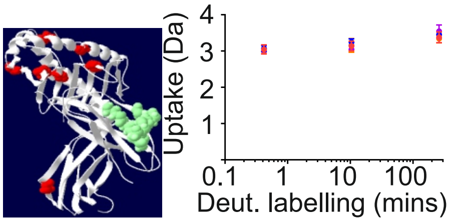

ca) ß2m 38-49

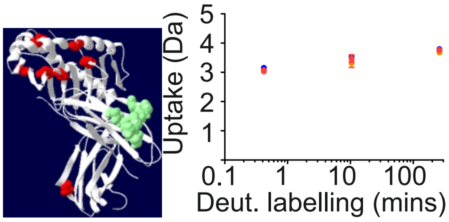

cb) ß2m 39-49

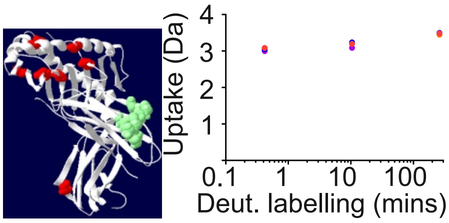

cc) ß2m 40-49

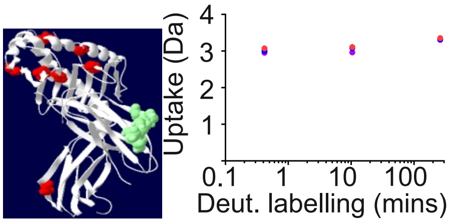

cd) ß2m 54-60

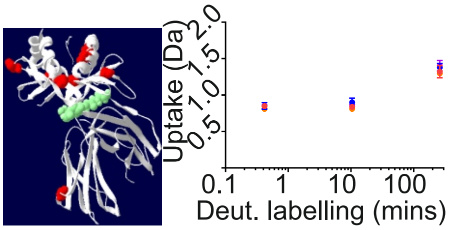

ce) ß2m 54-61

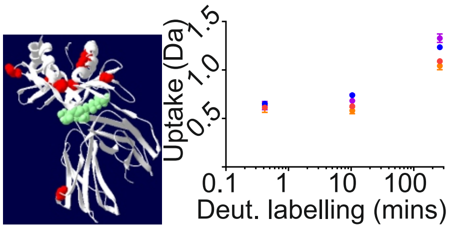

cf) ß2m 54-62

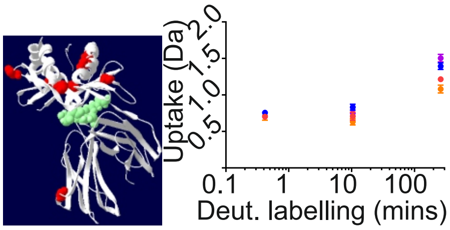

● 2 state, non-exposed ● 2 state, exposed ● 3 state, non-exposed ● 3 state, exposed

Supplementary figure 6

cg) ß2m 56-62

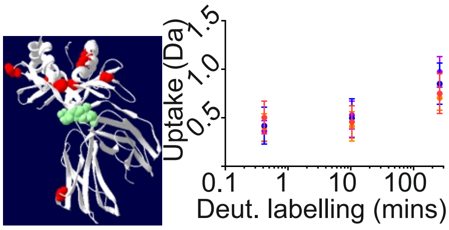

ch) ß2m 59-76

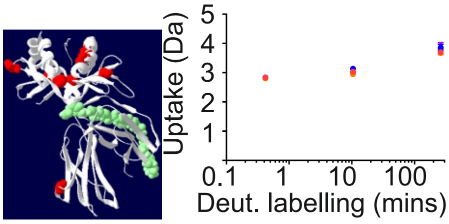

ci) ß2m 61-76

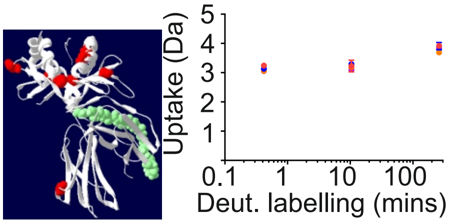

cj) ß2m 62-76

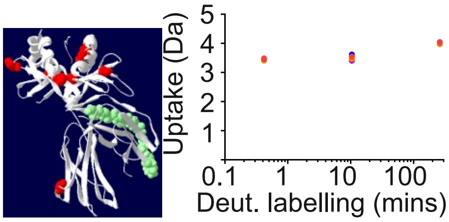

ck) ß2m 62-77

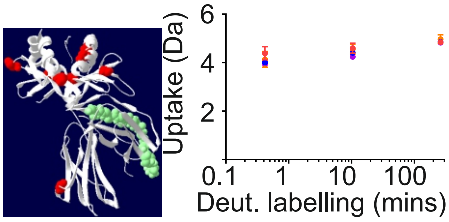

cl) ß2m 63-76

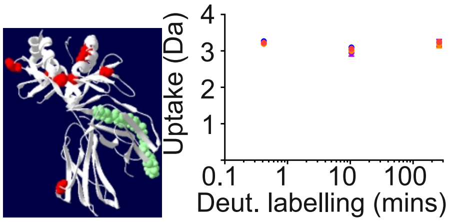

cm) ß2m 65-76

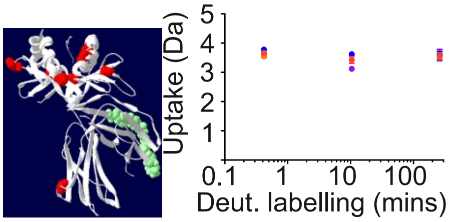

cn) ß2m 70-76

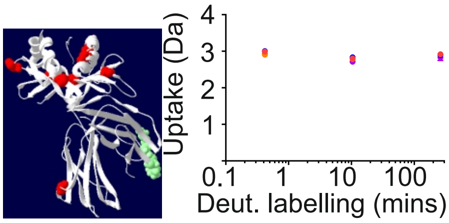

co) ß2m 77-91

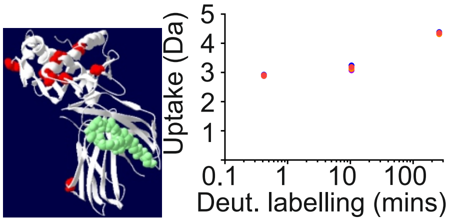

cp) ß2m 77-98

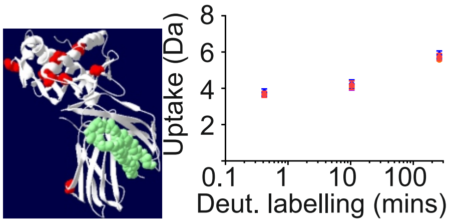

cq) ß2m 80-91

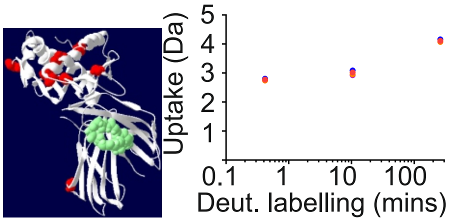

● 2 state, non-exposed ● 2 state, exposed ● 3 state, non-exposed ● 3 state, exposed

Supplementary figure 6 Comparison of deuterium uptake for polypeptides derived from BF2*19:01, in either the conditional ligand-loaded or peptide-receptive states, that were obtained from two experiments.

Charts comparing the uptake of deuterium for polypeptides derived from BF2*19:01, in either the conditional ligand-loaded or peptide-receptive states, that were obtained from two experiments. The results of the "three state" experiment (whose results are shown in figure 2, in which non-exposed samples were compared with either UV-exposed samples, or with UV exposed and peptide supplemented samples), were compared with the results of the "two state" experiment (whose results are shown in figures 4-10, in which non-exposed samples were compared with their UV-exposed counterparts). The uptake of deuterium after incubation in deuterated buffer for either 25 seconds, 10.4 minutes, or 4.34 hours is shown. Only those polypeptides derived from BF2*19:01 in the three state experiment which were also observed for both BF2 allotypes in the two state experiment are considered. Polypeptides were considered as being common to both allotypes based upon their location within the protein sequence. The mean average deuterium uptake is plotted for each exposure time and protein state, with the standard deviation that was observed between replicates represented using vertical error bars. For each polypeptide the indicated sequence is shown in green based on a homology model of the BF2*15:01-KRLIGKRY complex. Polymorphic residues are shown in red, or in orange when the polymorphism is located within the polypeptide of interest.
